# Comparison of Antioxidant and Antiproliferative Effects of Various Forms of Garlic and Ramsons

**DOI:** 10.3390/molecules28186512

**Published:** 2023-09-08

**Authors:** Paulina Furdak, Natalia Pieńkowska, Ireneusz Kapusta, Grzegorz Bartosz, Izabela Sadowska-Bartosz

**Affiliations:** 1 Laboratory of Analytical Biochemistry, Institute of Food Technology and Nutrition, College of Natural Sciences, University of Rzeszow, 4 Zelwerowicza Street, 35-601 Rzeszow, Poland; paulinaf2@o2.pl (P.F.); natalia.pien@gmail.com (N.P.); 2 Doctoral School, University of Rzeszow, 16C Rejtana Street, 35-959 Rzeszów, Poland; 3 Department of Food Technology and Human Nutrition, Institute of Food Technology and Nutrition, College of Natural Sciences, University of Rzeszow, 4 Zelwerowicza Street, 35-601 Rzeszow, Poland; ikapusta@ur.edu.pl; 4 Department of Bioenergetics, Food Analysis and Microbiology, Institute of Food Technology and Nutrition, College of Natural Sciences, University of Rzeszow, 4 Zelwerowicza Street, 35-601 Rzeszow, Poland; gbartosz@ur.edu.pl

**Keywords:** garlic, ramsons, antioxidant capacity, ovarian cancer cells, PEO1, SKOV3

## Abstract

Garlic is known to be rich in antioxidants, inhibit the proliferation of various cancer cells, and hamper cancer formation and growth, but various forms of garlic can differ greatly in these respects. This study aimed to compare the antioxidant properties of acetone, ethanol, and aqueous extracts of fresh Polish and Spanish garlic, black and granulated garlic, as well as fresh and dried ramsons. Extracts of black and granulated garlic showed the lowest total antioxidant capacity (TAC). The content of phenolic compounds correlated with TAC measured by ABTS^•^ decolorization and FRAP methods, and with the results of FRAP and DPPH^•^ decolorization assays. Garlic extracts inhibited the proliferation of PEO1 and SKOV3 ovarian cancer cells and, usually to a smaller extent, MRC-5 fibroblasts. PBS extracts of fresh Spanish garlic showed the highest potency for inhibition of proliferation of PEO1 cells (IC_50_ of 0.71 µg extract dry mass/100 µL medium). No significant correlation was found between the potency for inhibition of proliferation and the content of phenolics or flavonoids, confirming that phenolics are the main determinants of TAC but do not contribute significantly to the antiproliferative effects of garlic.

## 1. Introduction

Garlic (*Allium sativum* L., Amaryllidaceae) is one of the most commonly used spices worldwide. There are several incentives to consume garlic. Apart from the taste qualities, garlic is rich in antioxidants and compounds with beneficial health actions, such as antibiotic [1,2], antihypertensive [3,4], anti-rheumatic [5], hypoglycemic [6,7], and cholesterol-lowering effects [8,9]. It was also reported to have anti-parasite [10], anti-fungal [11,12], anti-bacterial [13,14], and anti-viral [15,16] properties. For these reasons, garlic can be categorized as both a food and a medicinal herb [17]. In particular, the anticancer effects of garlic have been reported by many authors [18]. Garlic extracts restrained the growth of human breast cancer MCF-7 cells [19,20,21], AGS and SGC-7901 gastric cancer cell lines [22], Colo205 [19,23], Caco-2 and HT-29 colon cancer cells, HepG2 human hepatic cancer cells [21], EJ human bladder cancer cells [24], and PC-3 human prostate cancer cells in vitro [21]. Treatment with fresh garlic extract (GE) significantly reduced the viability of leukemia cells collected from patients with childhood precursor-B acute lymphoblastic leukemia (ALL) [25]. Garlic oil inhibited the proliferation of human pancreatic carcinoma (AsPC-1, Mia PaCa-2, and PANC-1) cells [26]. Garlic was also found to show anticancer activity at the organismal level. Lung cancer risk in a Chinese population was found to be inversely associated with raw garlic intake [27]. High consumption of *Allium* vegetables, especially garlic, was associated with a lower risk of prostate cancer in men [28] and of esophageal cancer in both men and women [29]. Garlic intake was found to have a protective effect against colorectal cancer [30,31], although the evidence is inconsistent [32]. Moderate garlic intake was found to be inversely correlated with colorectal and renal cell carcinomas, whereas high intake had an inverse relationship with the incidence of colorectal, renal, oral, larynx, large bowel, and ovarian carcinomas [33]. Garlic intake was inversely associated with the risk of gastric cancer [29,33,34,35,36,37]. Aged garlic extract (AGE) suppressed the growth of colorectal adenoma [38], lessened the size and number of precancerous colorectal lesions [39], and suppressed the growth of tumors from sarcoma cells transplanted into the backs of ICR mice [40]. Oral administration of AGE to mice inhibited tumor growth and increased the activities of NK cells and killer cells in the spleen [40].

However, the properties of garlic can differ significantly depending on the cultivar, conditions of cultivation, drying conditions, and storage time and conditions [41,42]. The levels of soluble sugar were reported to decrease during storage, and the contents of total polyphenols and organosulfur compounds reached maximum values at 6 and 8 weeks, respectively, and then decreased significantly [43]. It was the reason for the comparison of Polish garlic, produced locally, and Spanish garlic, cultivated under different conditions and imported from Spain.

The drying process is one of the most effective methods used to reduce the moisture content of garlic in order to lengthen the shelf life of garlic [44]. Apart from fresh garlic, dried garlic is often consumed in granulated form, as its application for cooking is more convenient. Solid components are (about 3 times) more concentrated compared to fresh garlic, but many volatile compounds are lost, and others may be inactivated in the process of drying. The demand for dried garlic, in the form of slices, granules, or powder, has increased due to its widespread use as an ingredient in precooked foods and instant convenience foods [45].

Black garlic is obtained from fresh garlic (*Allium sativum*) that has been fermented for a period of time at a controlled high temperature (60–90 °C) under controlled high humidity (80–90%). When compared to fresh garlic, black garlic does not release a strong offensive flavor owing to the reduced content of allicin [46].

Ramsons (bear’s garlic) *Allium ursinum* L. has similar, but generally weaker, health benefits as cultivated garlic. Apart from the use of ramsons in folk medicine, it is tasty and can be eaten either raw or cooked. The bulbs are smaller and milder compared to *A. sativum*, but not only bulbs but also herbs of *A. ursinum*, both fresh and dried, are used and consumed [47,48].

Garlic is also interesting as a vegetable rich in antioxidants. Antioxidant properties of garlic may contribute to its anticancer properties, as reactive oxygen species are thought to participate in tumor initiation and progression [49]. Garlic was found to have the highest peroxy radical scavenging capacity estimated by the ORAC assay from among more than 20 common vegetables [50]. Heating decreased the total antioxidant capacity (TAC) of garlic, but the TAC of the heated brown garlic increased with the degree of browning to some extent [51]. For ramsons, the TAC of leaves is much higher than that of bulbs [52] and depends on the period of vegetation [53,54]. Organosulfur compounds were found to have negligible influence on the TAC of garlic and ramsons [53]. The role of phenolic compounds was found to “only have a partial influence” on the TAC of garlic and ramsons by some authors [53]; however, most authors attribute the TAC of garlic mostly to phenolic compounds [54,55]. 

When comparing the effects of extracts of various fruits and vegetables on the proliferation of ovarian cancer cells, we found an exceptional antiproliferation ability of aqueous garlic extracts. The proliferation of PEO1 and SKOV3 ovarian cancer cells was 50% inhibited by extracts from 1.11 and 3.33 μg of garlic per 100 μL medium, respectively [56]. The aim of the present study was to examine how this antiproliferative activity of garlic extracts varies within various garlic cultivars and preparations available commercially, and how it depends on the solvent used for extraction. 

The cytotoxic action of garlic is attributed mainly to allicin and other thiosulfinates, generated by alliinase upon crushing or other types of damage to garlic [57,58]. However, garlic is also rich in antioxidants, especially phenolic compounds, including flavonoids, among them quercetin derivatives [59,60], which contribute to its pro-health properties [2,61,62]. Therefore, the antioxidant capacity of various garlic preparations as well as their content of phenolic compounds and flavonoids were also compared. Fresh and dried ramsons *A. ursinum* were also included in the comparison.

## 2. Results

### 2.1. Antioxidant Capacity of Garlic Extracts

**Figure 1:**
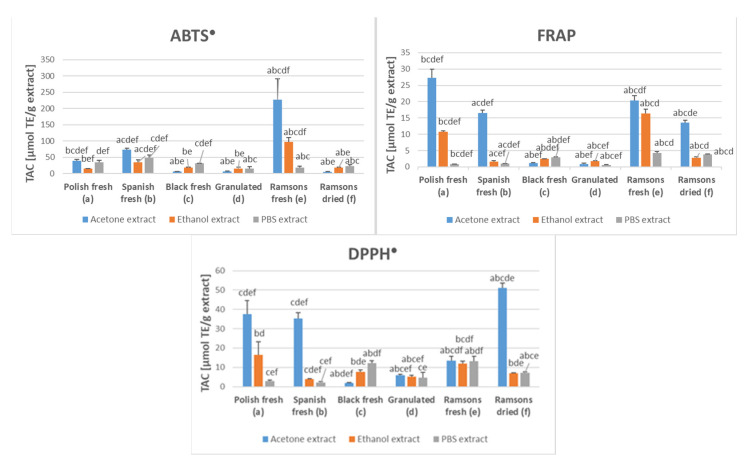
Total antioxidant capacity of PBS, ethanol, and acetone extracts of various garlic preparations. TE, Trolox equivalents. The letters above bars indicate the statistical significance of differences with respect to extracts of garlic types labeled with these letters (*p* < 0.05).

The total antioxidant capacity of acetone, ethanol, and PBS extracts of various garlic preparations showed considerable differences. Acetone extracts showed the highest TAC as estimated by all methods applied in fresh Spanish and Polish garlic compared to ethanol and PBS extracts, which was not the case for black and granulated garlic. In the ABTS^●^ decolorization assay, the highest TAC was found for acetone and ethanol extracts of fresh ramsons. The FRAP assay and the DPPH^●^ reduction assay showed the highest TAC for acetone extracts of fresh Polish garlic, fresh Spanish garlic, and fresh and dried ramsons (Figure 1).

**Figure 2:**
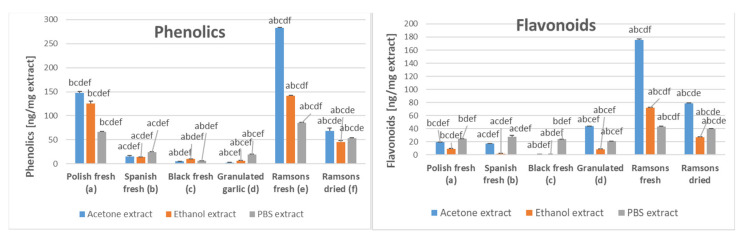
The content of phenolic compounds and flavonoids in PBS, ethanol, and acetone extracts of various garlic preparations. The letters above bars indicate the statistical significance of differences with respect to extracts of garlic types labeled with these letters (*p* < 0.05).

The content of phenolic compounds was the highest for the acetone extracts of Polish fresh garlic, fresh ramsons, and dried ramsons. All types of extracts of Spanish fresh garlic, black garlic, and granulated garlic had much lower content of phenolic compounds. Fresh and dried ramsons had a higher content of flavonoids than other garlic preparations in all types of extracts (Figure 2).

### 2.2. Garlic Content of Organosulfur Compounds

Six organosulfur compounds were identified in the extracts at amounts allowing for estimation of concentrations (Table 1; UPLC-PDA-MS chromatograms shown in the Supplementary Material). The content of these compounds in the ethanol and PBS extracts of *Allium sativum* is summarized in Table 2. No significant amounts of organosulfur compounds, allowing for a reliable analysis, were detected in the acetone extracts of *A. sativum* and in extracts of either fresh or dried *A. ursinum*.

### 2.3. Ramsons Content of Phenolics

Sixteen phenolic compounds were identified in ramsons in amounts allowing quantification (Table 3; UPLC-PDA-MS chromatograms shown in the Supplementary material). The content of phenolics in the ethanolic and PBS extracts of fresh and dried ramsons is given in Table 4. No amounts of phenolics sufficient for reliable analysis were found in the acetone extracts.

### 2.4. Inhibition of Cell Proliferation In Vitro by Garlic Extracts

Garlic extracts inhibited the proliferation of PEO1 and SKOV3 ovary cancer cells and, usually less efficiently, of MRC-5 fibroblasts. Acetone extracts of fresh Polish garlic and dried ramsons; ethanol extracts of fresh black garlic; and PBS extracts of fresh Spanish garlic, granulated garlic, and fresh ramsons were the most effective in hampering the proliferation of PEO1 cells. The proliferation of SKOV3 cells was the most inhibited by acetone extracts of fresh Polish and fresh Spanish garlic, and of dried ramsons, ethanol extracts of fresh black garlic and fresh ramsons, and PBS extracts of granulated garlic. The proliferation of MRC-5 fibroblast was hampered most efficiently by acetone extracts of fresh Polish and Spanish garlic, fresh and dried ramsons, ramsons, ethanol extracts of fresh black garlic, and PBS extracts of granulated garlic. In absolute terms, PBS extracts of fresh Spanish garlic showed the highest potency for inhibition of proliferation of PEO1 cells (IC_50_ of 0.71 µg/100 µL medium), acetone extracts of Polish garlic induced the highest inhibition of SKOV3 cells (IC_50_ of 6.0 µg/100 µL medium), while the proliferation of MRC-5 cells was the most efficiently inhibited by acetone extracts of fresh Polish garlic (IC_50_ of 6.0 µg/100 µL medium) (Table 5).

**Figure 3:**
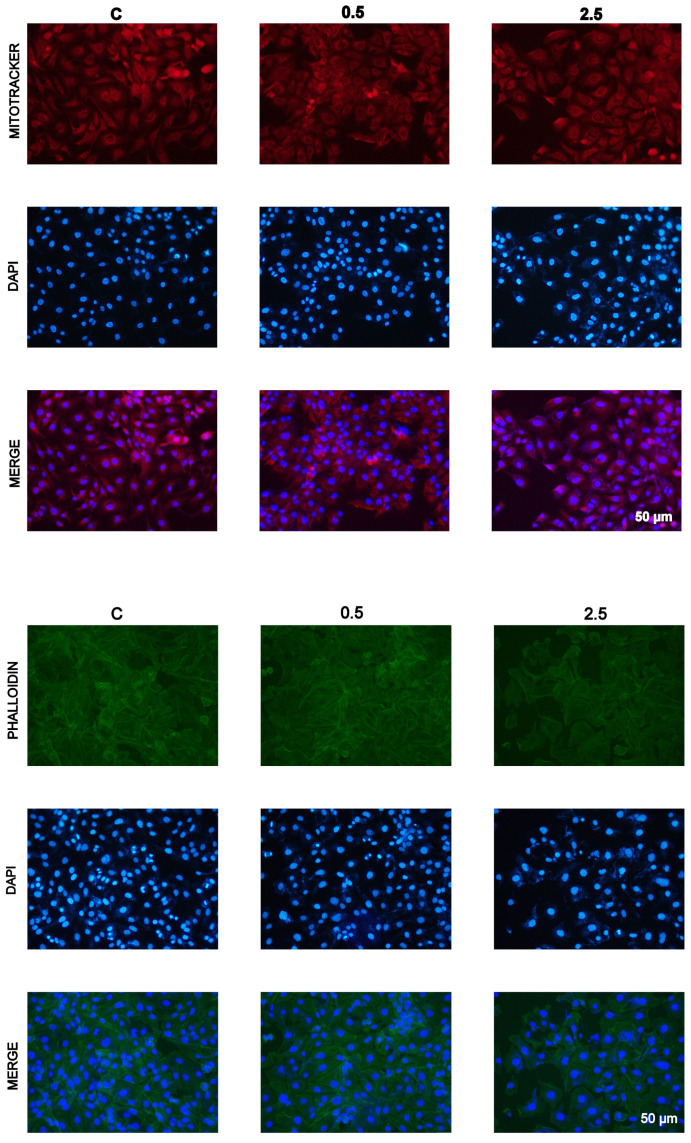
Mitotracker Deep Red FM and Atto-488-phalloidin staining of control (K) SKOV3 cells, and the cells treated with 0.5 μL/100 μL and 2.5 μL/100 μL of the ethanol extract of Spanish garlic/100 μL medium (8.7 and 43.6 μg of extract dry mass/100 μL medium, respectively).

Cell cultures were stained with Mitotracker Deep Red FM and Atto-488-phalloidin, which target intracellular mitochondrial network and the cytoskeletal actin, respectively. Microscopic examinations of cells treated with the ethanol extract of Spanish garlic showed decreased staining of both mitochondria and the cellular cytoskeletal actin network (Figure 3).

Associations between various parameters studied were evaluated by calculating respective correlation coefficients, taking into account all extracts of all forms of garlic studied (18 results for each parameter). There were strong, highly significant correlations between the content of total phenolics and flavonoids, between the content of phenolics and TAC estimated by the ABTS^•^ decolorization assay and by the FRAP assay as well as between the flavonoid content and TAC estimated by the ABTS^•^ decolorization assay. A weaker but still statistically significant correlation was revealed between the flavonoid content and TAC estimated by the FRAP assay. With respect to the relationships between various assays of TAC of garlic extracts, there was a statistically significant correlation between the results obtained by the ABTS^•^ decolorization assay and by the FRAP assay and an even stronger correlation between the results of the FRAP assay and of the DPPH• decolorization assay (Table 6). The correlations between the sum of concentrations of organosulfur compounds in ethanol and PBS extracts of the garlic forms studied and IC_50_ values of the extracts for PEO1, SKOV3, and MRC-5 cells were not significant (−0.46, −0.31, and −0.39, respectively) but it may be due to the low amount of data compared. The correlation between IC_50_ values for two ovarian cancer cell lines studied was high while those between IC_50_ values for ovarian cancer cell lines and MRC-5 fibroblasts were still statistically significant but lower.

## 3. Discussion

The results of this study demonstrate considerable differences in the antioxidant properties and inhibition of cell proliferation between extracts of different garlic cultivars and preparations. Extracts of fresh Polish garlic had a higher content of phenolic compounds than extracts of fresh Spanish garlic. Fresh black garlic and granulated garlic each had a low content of phenolics. Phenolic compounds are apparently the main contributors to the TAC of garlic extracts, and a significant correlation was found between the phenolic content and TAC measured by the ABTS^●^ decolorization assay and the FRAP assay. These results are in agreement with the data reported by other authors. Chen et al., comparing 43 various garlic cultivars, found correlation coefficients between total phenolic content and TAC estimated by DPPH reduction, FRAP, and CUPRAC methods of 0.68, 0.92, and 0.92, respectively, and between total flavonoid content and TAC estimated by DPPH reduction, FRAP, and CUPRAC methods of 0.25, 0.16, and 0.33, respectively [54]. Škrovánková et al. reported higher values of correlation coefficients from comparisons of a smaller number of cultivars (r_phenolics/TAC-ABTS_ = 0.97, r_phenolics/TAC-DPPH_ = 0.87) [55]. Extracts of ramsons, especially fresh ramsons, showed also a high phenolic content and high TAC. Extracts of dried ramsons showed especially high TAC in the DPPH^●^ decolorization assay, mainly dependent on hydrophobic antioxidants, indicating that acetone extraction of hydrophobic antioxidants from the dried ramsons is more efficient than from fresh ramsons, perhaps due to the lowering of acetone concentration by water present in fresh ramsons.

The values of TAC of garlic extracts obtained with the ABTS^●^ decolorization assay were significantly higher than those obtained with the FRAP assay and the DPPH assay. These results are understandable, taking into account the high reactivity of the ABTS^●^ radical [63] and steric hindrance problems in the reactions of some antioxidants with DPPH^●^ [64]. Interestingly, however, a high correlation was revealed between the results of the FRAP and DPPH^●^ decolorization assays.

A general conclusion resulting from this study is that fresh garlic is richer in antioxidants than black garlic, and ramsons is quite rich in antioxidants, as shown recently [46].

The strong inhibition of the proliferation of PEO1 cells by PBS extracts of fresh Spanish garlic is in general agreement with the results of our previous study [56] in which such extracts were used. We found previously 50% inhibition of the proliferation of PEO1 cells by a PBS extract from Spanish garlic at a concentration corresponding to 0.31 μg extract/100 μL (using a different batch of garlic). This finding is of interest, as the PBS extraction is the closest to the physiological conditions of garlic digestion and suggests a possible antineoplastic effect of garlic in vivo. The cytotoxic action of the extracts involves effects on the mitochondria and on the cytoskeletal actin filaments. 

Quantitative comparison of these results with the literature data on the cytotoxicity of garlic extracts for cancer cells in vitro is not easy since they are expressed in various ways as far as the extract concentrations are concerned. It was reported that crude aqueous extracts of garlic (0.25 μg /mL) reduced the proliferation of human cancer cell lines, such as colon (Caco-2), hepatic (Hep-G2), prostate (PC-3), and breast (MCF-7) as well as TIB-71 mouse macrophages by 44.9 ± 1.2%, 81.7 ± 2.9%, 90.5 ± 0.7%, 88.9 ± 0.1%, and 92.0 ± 0.6%, respectively. In this study, the extract was prepared by homogenization of seven garlic cloves with 1 mL of water, and the extract concentration was expressed in μg of the extract per mL of medium [21]. Aqueous/ethanolic garlic extracts (35 g garlic/25 mL 40% ethanol) inhibited by about 50% the proliferation of RPMI 8226 human myeloma cells at a dilution of 1:4000, the proliferation of JJN-3 plasma leukemia cells at a dilution of 1:1500, and the proliferation of DU 145 prostate cancer cells at a dilution of 1:750 [65]. The viability of HT29 human colon adenocarcinoma cells was decreased by 59% after 72 h exposure to garlic extract obtained by extraction of 10 g of dried garlic with 50 mL of 67% ethanol at a concentration of 10 mg extract/mL [66]. The proliferation of Caco-2 human colorectal adenocarcinoma cells was 50% inhibited by acetone/ethanol/ water extracts of garlic containing 2.06–3.60 μg of gallic acid equivalents of phenolic compounds/mL, depending on the garlic variety [67]. That study also illustrates the dependence of the cytotoxic activity of garlic on the origin of the material. Seven-day exposure of MCF-7 human breast adenocarcinoma cells to 100 ppm of aged garlic extract inhibited their proliferation by about 35% [68]. The estimated IC_50_ value of fresh garlic extracts (10 g of garlic/5 mL of DMEM-F12 cell medium) for the same cell line was 2.5 mg plant dry mass /mL [20]. Garlic extract inhibited the proliferation of human bladder cancer EJ cells by about 50% at the concentration of 800 μg/mL [24].

The cytotoxic effects of garlic are generally ascribed to the organosulfur compounds present in this plant, especially allicin [69,70,71]. Allicin inhibited the proliferation of human mammary (MCF-7) and colon (HT-29) cancer cells, with half maximal inhibitory concentration (IC_50_) values of 10–25 μM. Two of the three tested primary lines of human fibroblasts displayed a similar response to allicin (IC_50_ values of 16–40 μM), whereas the third line was almost unaffected by this compound [72]. Viability of human gastric adenocarcinoma SGC7901 cells was reduced by about 50% after 48 h incubation with 120 μg/mL allicin [73]. Allicin (50 nm) inhibited the proliferation of human SiHa cervical squamous cell carcinoma cells by 43% [74]. Allicin at a concentration of 5 μg/mL decreased the viability of human breast cancer MDA-MB-231 cells, while a similar effect was exerted by 17 μg/mL allicin on breast cancer MCF-7 cells [75]. Another study reported IC_50_ values of allicin for the inhibition of proliferation of HeLa, MCF-7, and L929 cells to be 0.15, 0.20, and 1.25 μg/mL, respectively [76]. Allicin at a concentration of 25 μg /mL inhibited the proliferation of SKOV3 cells at about 50% after 48 h [77].

Among other organosulfur compounds found in the extracts, there are reports on the cytotoxic effects of *S*-allylcysteine (SAC) and *S*-allylthiocysteine (SATC) on cancer cells. *S*-allylcysteine was reported to inhibit the proliferation of A2780 human ovarian cancer cell line cells with an IC_50_ of approximately 25 mmol/L for the 48 h treatment, and less than 6.25 mmol/L after 96 h [78], and 16.25 mmol/L and 5.25 mmol/L after 48 h and 96 h, respectively [79]. The IC_50_ values for the inhibition of proliferation of PC-3 and DU145 human prostate cancer cells by SAC were 2.2 and 4.6 mM, respectively [80]. The proliferation of human oral squamous cancer CAL-27 cells was inhibited by about 30% by 20 mM SAC [81]. The IC_50_ values of SAC for the inhibition of proliferation of hepatocellular carcinoma MHCC97L cells were about 33 mM and 22 mM on day 3 and day 4, respectively [82]. S-allylthiocysteine inhibited the proliferation of HEL and OCIM-1 human erythroleukemia cells, with IC_50_ values of 46 μM and 93 μM, respectively [83]. An about 50% inhibition of proliferation of HO8910PM and SKOV3 human ovarian cancer cells was evoked by 300 μM SATC, but the growth of the chemoresistant HO8910PM cell line was even enhanced by this concentration of SATC [84]. 

Though the relevance of in vitro experiments to in vivo situations is not straightforward, reduction of the tumor growth in experimental animals was demonstrated [38,39,40]. 

There were considerable differences in the antiproliferative activity between various types of extracts and various garlic forms. No significant correlation was found between the antiproliferative activity and the content of phenolics or flavonoids, confirming that phenolic compounds do not contribute significantly to the antiproliferative effect of garlic. The differences between the PBS extracts of fresh garlic of Spanish and Polish origin are especially striking, as the extracts of the Polish garlic showed very low antiproliferative activity in the concentration range studied, not allowing even for the extrapolation of an IC_50_ value. The correlation coefficients between the sum of concentrations of the organosulfur compounds studied in the ethanol and PBS extracts and IC_50_ values of the extracts were not significant (−0.46, −0.31, and −0.39 for PEO1, SKOV3, and MRC-5 cells, respectively), but apparently this was due to the low amount of data. Apart from genetic differences between the strains cultivated in both countries, the environmental factors as well as storage and transport conditions may also affect the alliin content and alliinase activity of garlic. Considerable differences in the alliin content were noted within one garlic variety (California Late) depending on the level of nitrogen fertilization and storage conditions [85]. These statements may increase, on one hand, consumers’ uncertainty with respect to the pro-health effects of garlic they are buying but, on the other hand, show that varieties and growth conditions can be found to optimize the desired biological effects of garlic. The correlation between the IC_50_ values of various garlic extracts for the two ovarian cancer cell lines was very high, while those between IC_50_ values for two ovarian cancer cell lines and IC_50_ values for MRC-fibroblasts were lower, which gives room for expectations that conditions can be found allowing for the inhibition of growth of cancer but not normal cells.

## 4. Materials and Methods

### 4.1. Reagents and Equipment

Dimethyl sulfoxide (DMSO) (CAS no: 67-68-5; cat. no. D2438; anhydrous, ≥99.9%), formaldehyde 4′,6-diamidine-2′-phenylindole dihydrochloride (DAPI; CAS no. 8718-90-3; cat.no. D9542), formaldehyde (CAS no. 50-00-0) solution (cat. no. F1635; 37 wt%), Triton X-100 (CAS no. 9036-19-5; cat. no. T8787), (±)-6-hydroxy-2,5,7,8-tetramethylchromane-2-carboxylic acid (Trolox) (CAS no. 53188-07-1; cat. no. 238813; purity ≥ 97%), Neutral Red (CAS no. 553-24-2; solution 0.33%, cat. no. N2889), 2,4,6-tris(2-pyridyl)-*s*-triazine (TPTZ; CAS no. 3682-35-7; cat. no. T1253; purity ≥ 98%), Folin-Ciocalteu’s phenol reagent (cat. no. F9252), acetic acid glacial (CAS no. 64-19-7; cat. no. A6283; 99%), gallic acid (CAS no. 149-91-7; cat. no. G7384; purity 97.5–102.5% (titration), Phalloidin-Atto 488 (cat. no. 49409) and Phosphate-Buffered Saline (PBS) (cat. no. PBS404.200) were obtained from Merck (Poznań, Poland). Ethanol (CAS no. 64-17-5; cat. no. 396480111; purity ≥ 99.8%), methanol (CAS no. 67-56-1, cat. no. 6219900110; purity ≥ 99.9%), and sodium acetate anhydrous (CAS no. 127-09-3, cat. no. BN60/6191; purity ≥ 99%) were purchased from Avantor Performance Materials Poland (Gliwice, Poland). 2,2′-Azino-bis (3-ethylobenzthiazoline-6-sulfonic acid) (ABTS) (CAS no. 504-14-6; cat. no. 10102946001; purity ≥ 99%) was provided by Roche (Warsaw, Poland). Acetone (CAS no. 67-64-1; cat. no. 111024800; purity ≥ 99.5%) and diethyl ether (CAS no. 60-29-7; cat. no. 113842106; purity ≥ 99.5%) were from Chempur (Piekary Śląskie, Poland). 2,2-Diphenyl-1-picrylhydrazyl (DPPH) (CAS no. 1898-66-4; cat. no. HY-112053; purity ≥ 99.13%), Tetrazolium Red (CAS no. 298-96-4; cat. no. HY-D0714; purity ≥ 99.85%), and iron(III) chloride (CAS no. 7705-08-0; cat. no. 451649; purity ≥ 99.99%) were provided by MedChemExpress (Monmouth Junction, NJ, USA). Aluminum chloride (AlCl₃) (CAS no. 7447-41-8; cat. no. 62476; purity ≥ 99.0%) was from Honeywell Fluka^TM^ (Charlotte, NC, USA). Distilled water was purified using a Milli-Q system (Millipore, Bedford, MA, USA). Mitotracker Deep Red FM (CAS no. 873315-86-7; cat. no. M22426) was provided by Thermo Scientific (Waltham, MA, USA).

The McCoy 5A cell culture medium (cat. no 22330-021), Roswell Park Memorial Institute (RPMI) medium + GlutaMAX (cat. no 72400-021), and Dulbecco’s phosphate-buffered saline (DPBS) (cat. no. 14040-117) were provided by Thermo Fisher Scientific (Waltham, MA, USA). The fetal calf serum (FBS) (cat. no. S1813), penicillin–streptomycin solution (cat. no. L0022), trypsin–EDTA solution (10×) (cat. no. X0930), as well as phosphate-buffered saline without Ca^2+^ and Mg^2+^ (cat. no. P0750) were purchased from Biowest (Nuaillé, France).

The 75 cm^2^ cell culture flasks (cat. no. 156499) and 96-well white plates (cat. no. 165306) were from Thermo Fisher Scientific (Waltham, MA, USA). The transparent 96-well cell culture plates (cat. no 655180) were provided by Greiner (Kremsmünster, Austria). Other sterile cell culture materials were purchased from Nerbe (Winsen, Germany).

Absorptiometric measurements were carried out using a Spark multimode plate reader (Tecan Group Ltd., Männedorf, Switzerland).

### 4.2. Garlic Samples

Garlic samples or preparations available commercially in April 2023 in Rzeszów, podkarpackie voivodeship, South-Eastern Poland, were used. Fresh garlic (*Allium sativum* L.) bulbs grown in Poland and Spain, granulated garlic (McCormick, Poland), black garlic (Farm of Marian Lato, Poland), and herb of *Allium ursinum* L. (To Naturalne, Poland) were purchased in local grocery shops. Fresh wild garlic came from the first author’s own cultivation (village of Terka, county of Solina, podkarpackie) and was collected in April 2023.

### 4.3. Preparation of Garlic Extracts

A portion of cut garlic cloves (about 5 g), herbs, or granulate was homogenized with 9-volume portions of PBS, acetone, or ethanol (96%). The prepared samples were mixed with the appropriate solvent for 10 min on ice, frozen, thawed, and centrifuged at 4000 rpm for 15 min. The supernatants obtained by centrifugation were pooled and aliquoted into 2 mL Eppendorf tubes. The samples were frozen at −80 °C. Fractions of extracts were dried at 105 °C until obtaining constant weight to determine the contents of solids. The results of TAC, phenolic and flavonoid content, and cytotoxicity are expressed in relation to the dry mass of the extracts.

### 4.4. Assays of Antioxidant Activity

#### 4.4.1. ABTS^•^ Decolorization Assay

A modified [86] assay of Re et al. [87] was used. Briefly, 200-μL aliquots of ABTS^●^ solution diluted with PBS to a concentration providing absorbance of 1.0 at 734 nm were pipetted to wells of a 96-well plate and added with 0–10 μL of the extracts or 0.5 mM Trolox, shaken, and left for 30 min at room temperature (21 ± 1 °C). Then, the decrease in absorbance was read.

#### 4.4.2. DPPH^•^ Scavenging Assay

The assay was conducted as described previously [88]. Briefly, aliquots (200 µL) of 0.3 mM 2,2-diphenyl-1-picrylhydrazyl (DPPH^•^) solution in methanol were added with various volumes (0–50 μL) of garlic extracts or 1 mM Trolox and incubated for 30 min in the dark at ambient temperature. The decrease in absorbance at 517 nm was then measured.

#### 4.4.3. FRAP Assay

A modified procedure of Benzie and Strain [89] was employed. Briefly, 200 μL aliquots of the freshly prepared working solution (0.3 M acetate buffer, pH 3.6/10 mM TPTZ in 40 mM HCl/20 mM FeCl_3_; 10:1:1) were pipetted into wells of a 96-well plate and added with various volumes (0–50 μL) of the extracts or 1 mM Trolox, mixed and incubated at room temperature for 30 min. Afterward, absorbance was measured at 593 nm against a reagent blank.

#### 4.4.4. Calculation of Antioxidant Capacity

The antioxidant capacity of garlic and ramson extracts was calculated by comparing the slopes of dependencies of absorbance changes on the concentrations of Trolox and extracts as described previously [86] using the formula:

Antioxidant activity = (slope of dependence of absorbance change on the volume of extract)/(slope of dependence of absorbance change on the amount of Trolox) and expressed in μmoles of Trolox equivalents (TE) per g of extracted material.

### 4.5. Estimation of Polyphenol Content

The polyphenol content of the extracts was estimated using the Folin–Ciocalteu reagent [90] and expressed in gallic acid equivalents (GAE).

### 4.6. Estimation of Flavonoid Content

The content of flavonoids was estimated with aluminum chloride following the procedure of Sulaiman and Balachandran [91].

### 4.7. Determination of Organosulfur Compounds by UPLC-PDA-MS/MS

Determination of organosulfur compounds was carried out using a UPLC equipped with a binary pump, column and sample manager, photodiode array detector (PDA), and tandem quadrupole mass spectrometer (TQD) with electrospray ionization (ESI) source working in negative mode (Waters, Milford, MA, USA), as described elsewhere [92]. Separation was performed using the UPLC BEH C18 column (1.7 µm, 100 mm × 2.1 mm, Waters) at 50 °C, at a flow rate of 0.35 mL/min. The injection volume of the samples was 5 µL. The mobile phase consisted of water (solvent A) and 40% acetonitrile in water, *v*/*v* (solvent B). The following parameters were used: capillary voltage of 3500 V; con voltage of 30 V; con gas flow of 100 L/h; source temperature of 120 °C; desolvation temperature of 350 °C; and desolvation gas flow rate of 800 L/h. Polyphenolic identification and quantitative analyses were performed on the basis of the mass-to-charge ratio, retention time, specific PDA spectra, fragment ions, and comparison of data obtained with commercial standards and literature findings. The analyses were performed in two replications.

### 4.8. Cell Culture

Two human ovarian cancer cell lines (SKOV3 and PEO1) and a normal human fibroblast MRC-5 cell line were used. The SKOV3 (HTB-77) and MRC-5 (CCL-171) cells were purchased from the American Type Culture Collection (ATCC). The PEO1 (10032308) cells were obtained from the European Collection of Authenticated Cell Cultures (ECACC).

The SKOV3 cells were cultured in McCoy’s 5A medium, the PEO1 cells in RPMI + GlutaMAX medium, and the MRC-5 cells in DMEM + GlutaMAX. All the media contained 10% heat-inactivated FBS and 1% *v*/*v* penicillin/streptomycin solution. The cells were cultured at 37 °C under 5% carbon dioxide and 95% humidity. They were passaged after reaching about 85% confluence. The Trypan Blue exclusion test was used for the evaluation of cell viability. A Thoma hemocytometer (Superior Marienfeld, Lauda-Königshofen, Germany) was used for cell counting.

### 4.9. Estimation of Cytotoxicity

The PBS extracts were added to the appropriate culture media at the indicated proportions. The media were then sterilized by filtration through 0.22 μm filters. Acetone and ethanol extracts were evaporated in wells of a multiwell plate, which were then added with the cell medium and incubated on a shaker for 1 h so the medium did not contain the solvents.

The cells were seeded in the wells of a 96-well plate. The initial density was a density of 1 × 10^4^ cells/well (SKOV3), 1.5 × 10^4^ cells/well (PEO1), or 7.5 × 10^3^ cells/well (MRC-5). The cells were allowed to grow for 24 h, then the medium was aspirated, 100 μL of extract-containing medium was added, and the cells were incubated for 24 h. Control cells received a medium containing no additives. Finally, the media were aspirated and replaced with a 2% Neutral Red solution (100 μL). The cells were incubated at 37 °C for 1 h, washed with PBS, fixed, and extracted with 50% ethanol and 49% H_2_O/1% glacial acetic acid (100 μL) at room temperature for 20 min with shaking (700 rpm). The absorbance of the extracts was measured at 540 nm against 620 nm. The assay was performed in six parallel samples.

### 4.10. Staining with Atto-488-Phalloidin, Mitotracker, and DAPI 

The cells were seeded at a density of 7.5 × 10^3^ cells/well in a 96-well transparent plate and allowed to adhere for 24 h. Then the cells were treated with Spanish garlic ethanol extract (0.5, 2.5, and 5 µL per 100 µL medium). After 24 h, the medium was aspirated, and 500 nM solution of Mitotracker Deep Red FM in PBS (100 µL) was added to each well. The plate was incubated for 1 h in a CO_2_ incubator. Afterward, the cells were washed with PBS (100 μL/well) and fixed with 3.7% formaldehyde (100 μL/well) for 15 min. Finally, the cells were washed with 200 μL/well of PBS, permeabilized with 100 μL/well of 0.1% Triton X-100 for 15 min, and washed with PBS (200 μL/well). 

Alternatively, following the incubation with the extract, the cells were stained with Atto-488-phalloidin working solution according to the manufacturer’s protocol (100 μL/well) for 60 min.

After the staining, the cells were washed with PBS, 100 µL of 600 nM solution of DAPI in PBS was added to each well, and the cells were incubated at room temperature for 1 h and inspected under a fluorescence microscope.

### 4.11. Statistics

All measurements were conducted at least three times on different preparations, each time at least in triplicate. In the TAC measurements, the dependencies of absorbance changes on the concentration of extracts were linear, so the slopes were calculated using the REGLINP function (Excel). The statistical significance of differences was evaluated using the two-tailed Student’s “*t*” test (Excel). 

To estimate the differences between the viabilities of cells treated with garlic extract and control cells, the Kruskal–Wallis test (n ≥ 6 independent experiments) was performed. Always, *p* ≤ 0.05 was considered as statistically significant. The statistical analysis of the data was performed using the STATISTICA software package (version 13.1, StatSoft Inc., 2016, Tulsa, OK, USA).

## 5. Conclusions

Considerable differences were found in the content of phenolic and flavonoid compounds, and TAC and antiproliferative activity with respect to ovarian cancer cells between various types of extracts and various garlic cultivars and preparations. Black garlic and granulated garlic had lower contents of phenolics and lower TAC than fresh Polish and Spanish garlic and fresh and dried ramsons. Extracts of black garlic had the lowest antiproliferation activity. Strong, statistically significant correlations were found between the content of total phenolics and flavonoids, as well as between the content of phenolics and TAC estimated by the ABTS^•^ decolorization assay and by the FRAP assay, and between the flavonoid content and TAC estimated by the ABTS^•^ decolorization assay.

## Figures and Tables

**Table 1:** Individual organosulfur compounds identified by UPLC-PDA-MS/MS in *A. sativum*.

Compound	R_t_ [min]	[M-H]^+^ *m*/*z*	
		MS	MS/MS
1. *S*-Ethylcysteine sulfoxide	1.83	166	88
2. γ-Glu-allylcysteine	2.34	291	145
3. γ-Glu-propenylcysteine	2.75	291	145
4. γ-Glu-allylthiocysteine	3.66	323	145
5. Allicin	6.19	163	73
6. *S*-allylcysteine	7.05	163	146

**Table 2:** Content of organosulfur compounds in various garlic extracts.

Compound	Garlic	Ethanol Extract	PBS Extract
*S*-Ethylcysteine sulfoxide	Polish fresh	-	0.56 ± 0.02
	Spanish fresh	1.00 ± 0.04	2.31 ± 0.10
	Black fresh	-	9.78 ± 0.41
	Granulated	-	-
γ-Glu-allylcysteine	Polish fresh	-	1.29 ± 0.07
	Spanish fresh	11.26 ± 0.62	17.97 ± 0.99
	Black fresh	-	-
	Granulated	-	23.68 ± 1.30
γ-Glu-propenylcysteine	Polish fresh	3.27 ± 0.11	14.06 ± 0.46
	Spanish fresh	3.64 ± 0.12	5.25 ± 0.17
	Black fresh	-	-
	Granulated	-	92.25 ± 3.04
γ-Glu-allylthiocysteine	Polish fresh	-	-
	Spanish fresh	2.41 ± 0.11	5.59 ± 0.25
	Black fresh	-	-
	Granulated	-	12.49 ± 0.55
Allicin	Polish fresh	7.24 ± 0.46	34.75 ± 2.23
	Spanish fresh	19.35 ± 1.24	40.43 ± 2.59
	Black fresh	-	-
	Granulated	-	47.72 ± 3.05
*S*-allylcysteine	Polish fresh	4.97 ± 0.14	4.80 ± 0.13
	Spanish fresh	7.65 ± 0.21	7.40 ± 0.20
	Black fresh	-	-
	Granulated	-	4.97 ± 0.14
Total	Polish fresh	15.48 ± 0.49	55.46 ± 2.28
	Spanish fresh	45.31 ± 1.41	78.95 ± 2.80
	Black fresh	-	9.78 ± 0.41
	Granulated	-	176.14 ± 4.53

-, trace amounts.

**Table 3:** Individual phenolic compounds identified by UPLC-PDA-MS/MS in *A. ursinum*.

Compound	R_t_ [min]	λ_max_ [nm]	[M-H]^+^ *m*/*z*	
			MS	MS/MS
Kaempferol 3-*O*-rutinoside-7-*O*-glucoside	1.83	264, 347	166	88
Coumaric acid glucoside	2.34	312	291	145
Kaempferol 3-*O*-glucoside-7-*O*-glucoside	2.75	264, 345	291	145
Kaempferol (acetyl)-glucoside-rhamnoside-glucoside isomer I	3.66	264, 347	323	145
Kaempferol (acetyl)-glucoside-rhamnoside-glucoside isomer II	6.19	264, 346	163	73
Kaempferol (acetyl)-glucoside-rhamnoside-glucoside isomer III	7.05	264, 338	163	145.73
Kaempferol rutinoside-coumaroyl-glucoside-glucoside isomer I	4.46	264, 315	1063	593, 447, 285
Kaempferol rutinoside-coumaroyl-glucoside-glucoside isomer II	4.56	266, 316	1063	593, 447, 285
Kaempferol 3-*O*-rutinoside	4.66	264, 338	593	285
Kaempferol rutinoside-coumaroyl-glucoside isomer I	5.05	266, 316	901	593, 447, 285
Kaempferol rutinoside-coumaroyl-glucoside isomer II	5.12	267, 316	901	593, 447, 285
Kaempferol 3-*O*-rutinoside acetyl derivative	5.27	264, 331	635	593, 285
Kaempferol rutinoside-coumaroyl-glucoside isomer III	5.36	264, 316	901	593, 447, 285
Kaempferol rutinoside-coumaroyl-glucoside isomer acetyl derivat. I	5.60	267, 315	943	901, 593, 285
Kaempferol rutinoside-coumaroyl-glucoside acetyl derivative II	5.72	264, 317	943	901, 593, 285
Kaempferol-rutinoside-feruloyl-glucoside acetyl derivative	5.77	264, 338	973	797, 593, 285

**Table 4:** Content of phenolic compounds in extracts of fresh and dry ramsons [μg/mL].

Compound	Fresh		Dry	
	Ethanol Extract	PBS Extract	Ethanol Extract	PBS Extract
Kaempferol 3-*O*-rutinoside-7-*O*-glucoside	15.30 ± 0.79	44.12 ± 0.82	52.01 ± 0.97	373.9 ± 6.99
Coumaric acid glucoside	3.81 ± 0.15	1.31 ± 0.07	2.00 ± 0.10	53.96 ± 2.79
Kaempferol 3-*O*-glucoside-7-*O*-glucoside	4.20 ± 0.07	13.64 ± 0.54	23.24 ±0.92	94.34 ± 3.74
Kaempferol (acetyl)-glucoside-rhamnoside-glucoside isomer I	3.88 ± 0.07	13.77 ± 0.21	9.03 ±0.14	92.77 ± 1.45
Kaempferol (acetyl)-glucoside-rhamnoside-glucoside isomer II	5.37 ± 0.20	10.40 ± 0.18	18.59 ± 0.33	157.7 ± 2.77
Kaempferol (acetyl)-glucoside-rhamnoside-glucoside isomer III	0.44 ± 0.01	2.16 ± 0.08	2.86 ± 0.11	20.06 ± 0.76
Kaempferol rutinoside-coumaroyl-glucoside-glucoside isomer I	1.64 ± 0.02	7.21 ± 0.22	9.73 ± 0.29	15.05 ± 0.45
Kaempferol rutinoside-coumaroyl-glucoside-glucoside isomer II	2.32 ± 0.11	16.18 ± 0.21	19.56 ± 0.25	73.45 ± 0.94
Kaempferol 3-*O*-rutinoside	4.10 ± 0.01	4.81 ± 0.23	5.71 ± 0.27	39.52 ± 1.88
Kaempferol rutinoside-coumaroyl-glucoside isomer I	1.28 ± 0.03	6.13 ± 0.01	9.14 ± 0.02	7.15 ± 0.02
Kaempferol rutinoside-coumaroyl-glucoside isomer II	2.91 ± 0.17	20.20 ± 0.40	29.60 ± 0.59	41.26 ± 0.82
Kaempferol 3-*O*-rutinoside acetyl derivative	1.14 ± 0.00	1.99 ± 0.11	9.31 ± 0.53	73.30 ± 4.17
Kaempferol rutinoside-coumaroyl-glucoside isomer III	2.50 ± 0.02	4.16 ± 0.01	8.61 ± 0.03	10.69
Kaempferol rutinoside-coumaroyl-glucoside isomer acetyl derivative I	0.41 ± 0.00	3.18 ± 0.02	7.63 ± 0.05	30.78
Kaempferol rutinoside-coumaroyl-glucoside acetyl derivative II	1.96 ± 0.04	5.00 ± 0.06	4.65 ± 0.06	14.32
Kaempferol-rutinoside-feruloyl-glucoside acetyl derivative	2.14 ± 0.02	0.59 ± 0.01	1.62 ± 0.04	27.89
Total	51.83 ± 0.54	151.5 ± 1.60	213.2 ± 4.59	1126 ± 27.46

**Table 5:** IC_50_ values of PBS, ethanol, and acetone extracts of various garlic preparations [μg dry extract/100 μL medium].

Garlic/Extract/Cell Line		PEO1	SKOV3	MRC-5
Polish fresh	Acetone extract	6.7	6.0	7.2
	Ethanol extract	12.0	12.4	19.2
	PBS extract	ND	21.8	121.5
	Acetone extract	12.1	39.0	24.1
Spanish fresh	Ethanol extract	40.1	134.4	453.7
	PBS extract	0.71	61.6	42.0
	Acetone extract	420.5	650.4	696.4
Black fresh	Ethanol extract	113.0	127.2	71.7
	PBS extract	341.6	581.9	2159
	Acetone extract	51.7	71.7	832.8
Granulated	Ethanol extract	78.2	45.0	1166
	PBS extract	3.8	33.7	125.7
	Acetone extract	8.1	21.1	14.0
Ramsons fresh	Ethanol extract	12.2	13.0	15.8
	PBS extract	6.2	29.2	29.4
Ramsons dried	Acetone extract	12.2	15.3	17.7
	Ethanol extract	50.3	56.3	70.4
	PBS extract	32.2	197.2	ND

ND, could not be determined.

**Table 6:** Values of correlation coefficients between the parameters studied.

Correlation Coefficient	Flavonoid Content	TAC/ABTS^●^	TAC/FRAP	TAC/DPPH^●^	IC_50_/PEO1	IC_50_/SKOV3	IC_50_/MRC-5
Phenolic content	0.78 ***	0.77 ***	0.74 ***	0.27	−0.38	−0.39	−0.44
Flavonoid content		0.81 ***	0.55 *	0.05	−0.23	−0.21	−0.23
TAC/ABTS^●^			0.55 *	0.05	−0.23	−0.21	−0.23
TAC/FRAP				0.73 **	−0.35	−0.35	−0.38
TAC/DPPH^●^					−0.28	−0.27	−0.26
IC_50_/PEO1						0.97 ***	0.69 **
IC_50_/SKOV3							0.69 **

* *p* < 0.05; ** *p* < 0.01; *** *p* < 0.001.

## Data Availability

Data are available from the corresponding author upon reasonable request.

## Supplementary Materials

The following supporting information can be downloaded at: https://www.mdpi.com/article/10.3390/molecules28186512/s1.

## Sample Availability

Samples of the materials are available upon request.
