# Supplementary material for: Comparison of Antioxidant and Antiproliferative Effects of Various Forms of Garlic and Ramsons
Source: Molecules. 2023 Sep 8;28(18):6512. doi: 10.3390/molecules28186512 (PMC10538172; doi:10.3390/molecules28186512)
Supplement: Supplementary file 1 [file molecules-28-06512-s001.zip › molecules-2568539-supplementary.pdf]

Supplementary Material S1

UPLC-PDA-MS chromatograms of organosulfur compounds found in *A. sativum*. Numbers of compounds as given in Table 1.

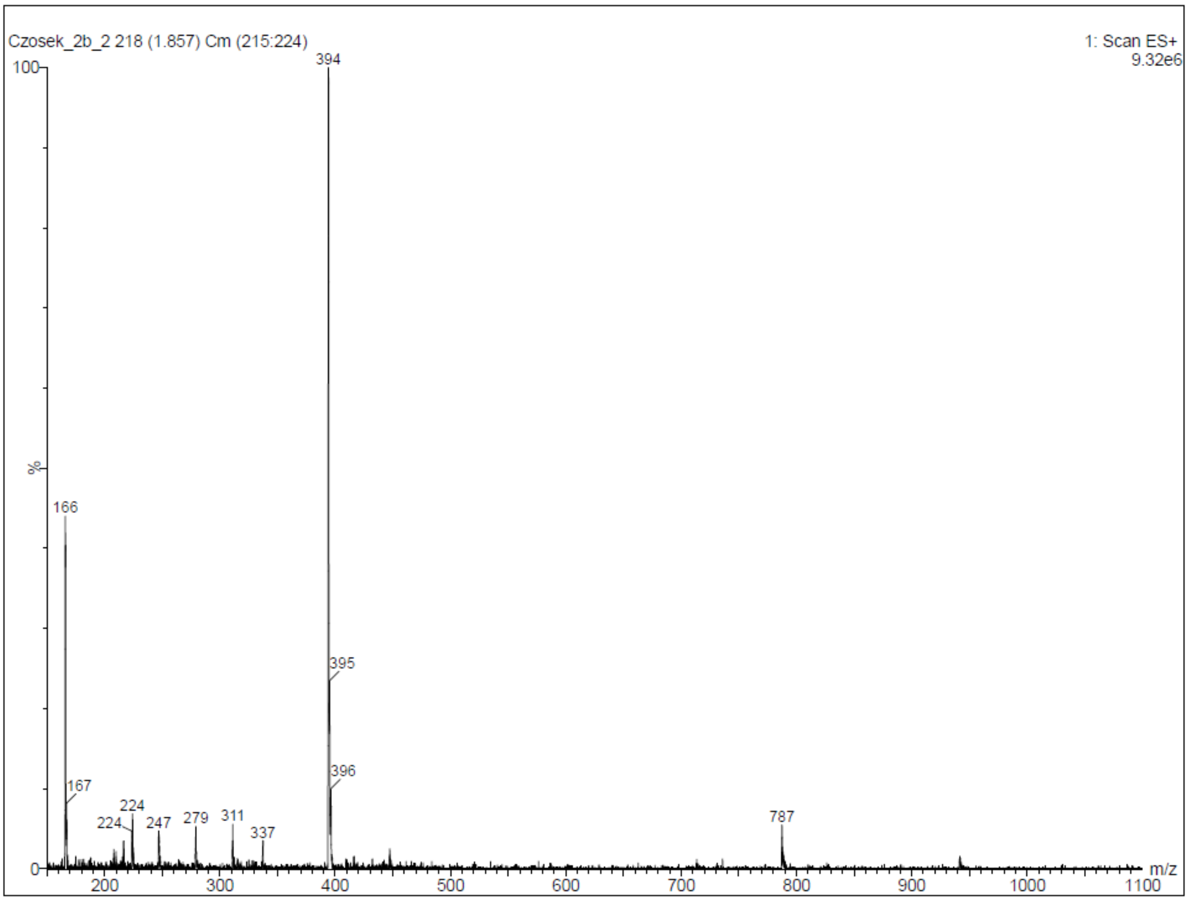

Compound 1

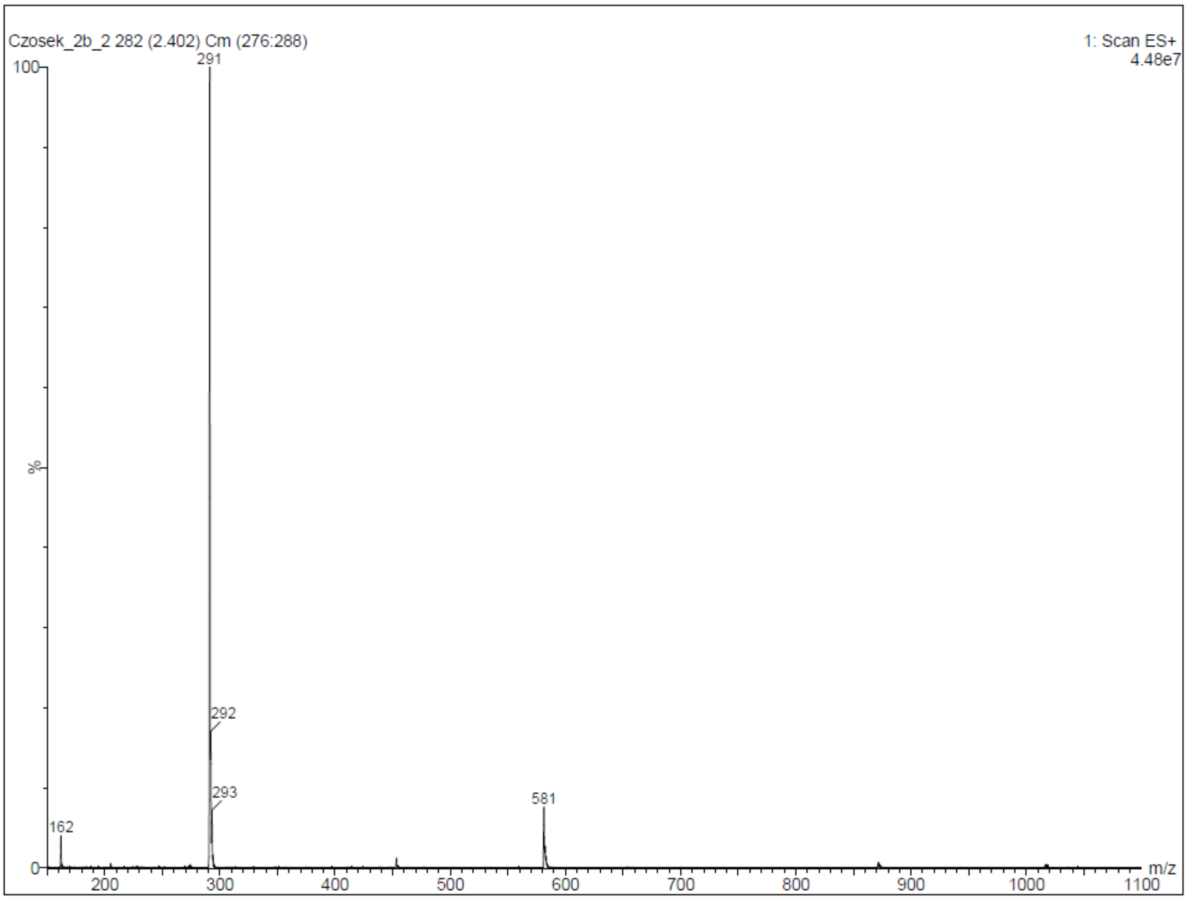
Czosek_2b_2 282 (2.402) Cm (276:288)
1: Scan ES+
4.48e7
100
%
0
291
292
293
162
581
200
300
400
500
600
700
800
900
1000
1100
m/z

Compound 2

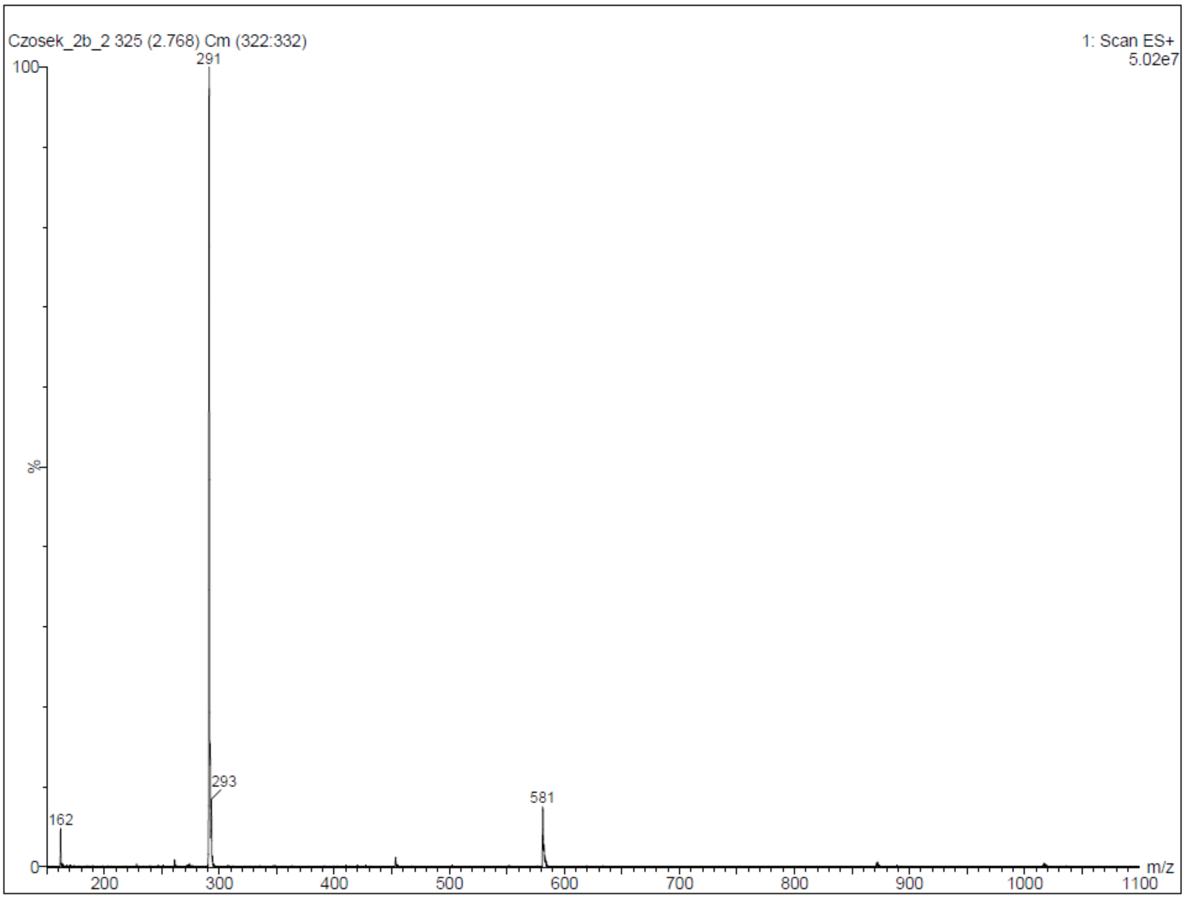
Czosek_2b_2 325 (2.768) Cm (322:332)
1: Scan ES+
5.02e7
100
%
0
291
293
162
581
200
300
400
500
600
700
800
900
1000
1100
m/z

Compound 3

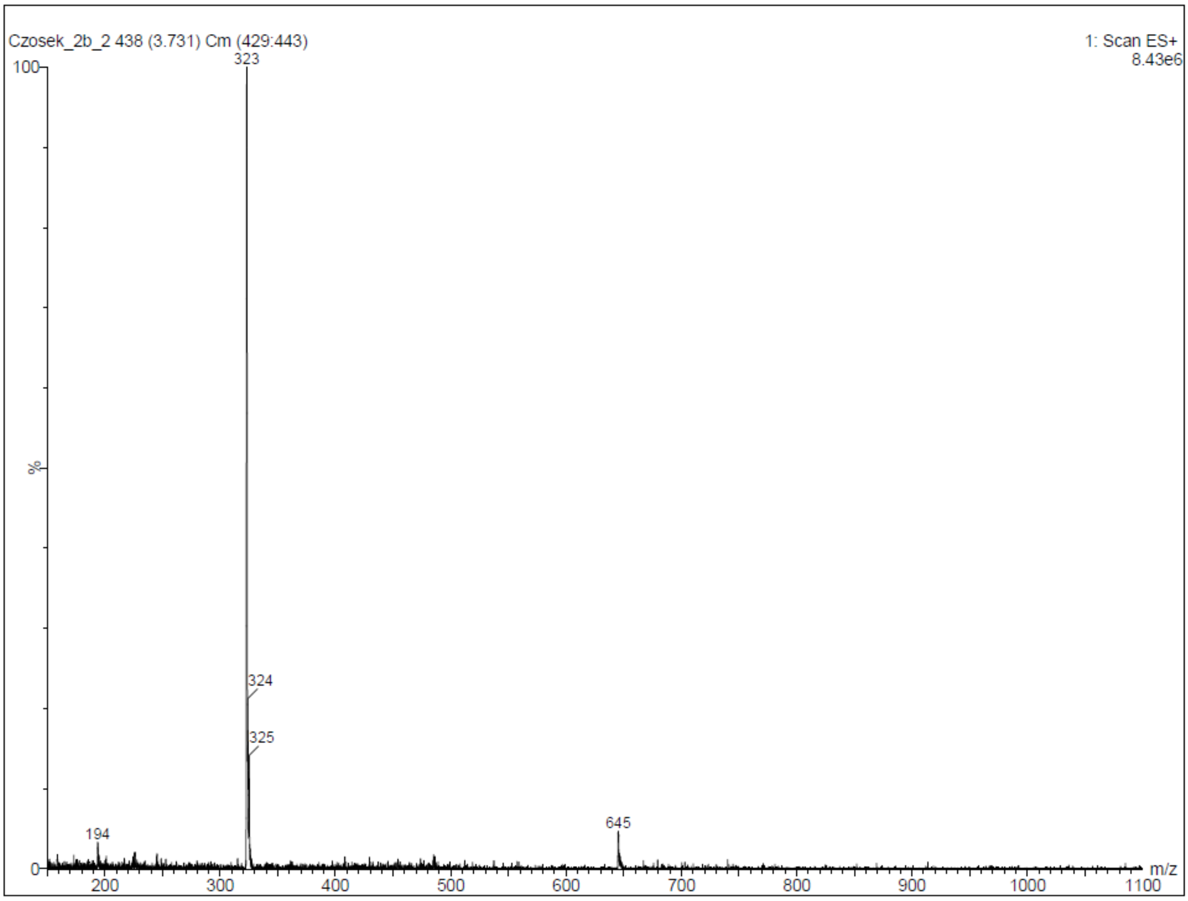

Czosek_2b_2 438 (3.731) Cm (429:443)
1: Scan ES+
8.43e6
100
%
0
323
324
325
194
645
m/z
200
300
400
500
600
700
800
900
1000
1100

Compound 4

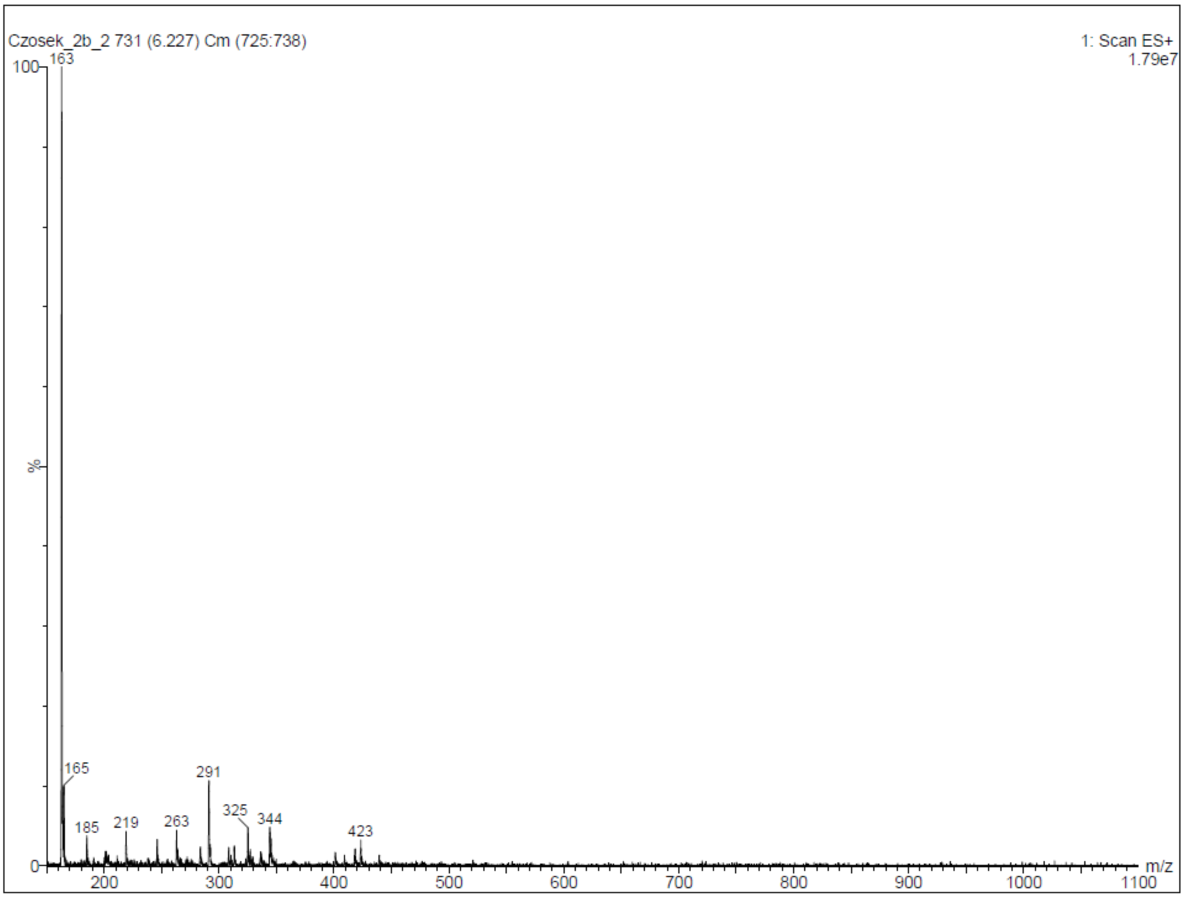

Czosek_2b_2 731 (6.227) Cm (725:738)
1: Scan ES+
1.79e7
100
163
%
165
291
185
219
263
325
344
423
0
m/z
200
300
400
500
600
700
800
900
1000
1100

Compound 5

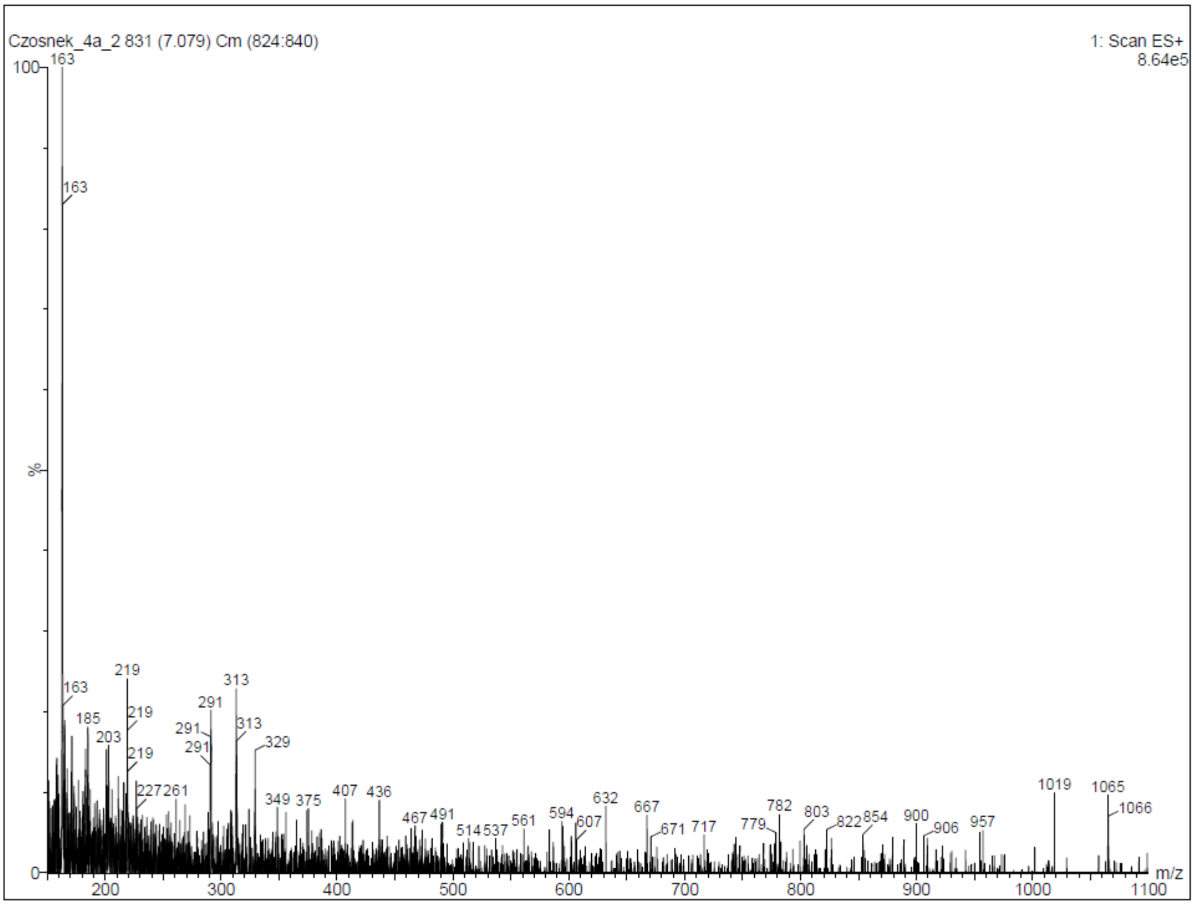
Czosnek_4a_2 831 (7.079) Cm (824:840)
1: Scan ES+
8.64e5
%
m/z
163
219
313
291
329
185
203
227
261
349
375
407
436
467
491
514
537
561
594
607
632
667
671
717
779
782
803
822
854
900
906
957
1019
1065
1066

Compound 6

Supplementary Material S2

UPLC-PDA-MS-MS spectra of phenolic compounds found in *A. ursinum*. Numbers of compounds as given in Table 3.

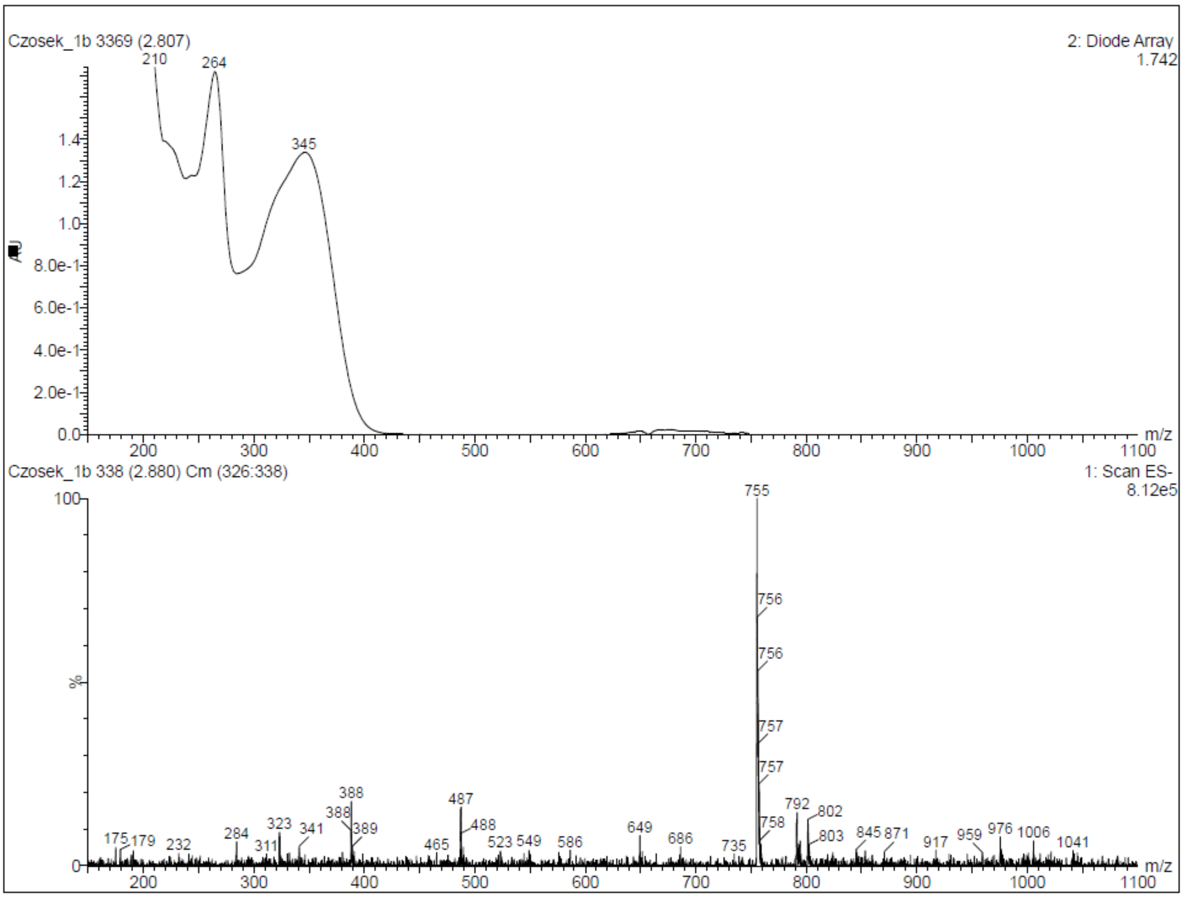

Compound 1

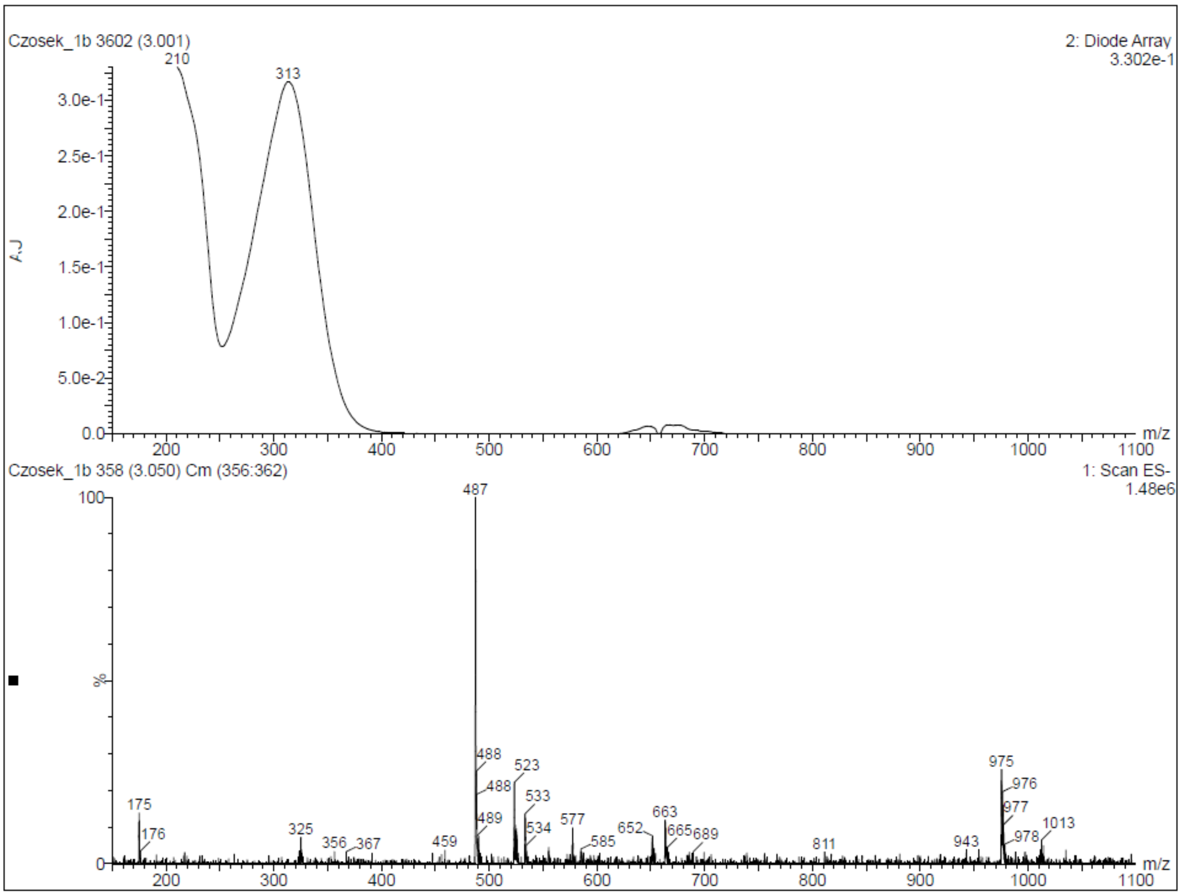
Czosek_1b 3602 (3.001)
2: Diode Array
3.302e-1
210
313
AU
m/z
Czosek_1b 358 (3.050) Cm (356:362)
1: Scan ES-
1.48e6
487
488
488
489
523
533
534
577
585
652
663
665
689
175
176
325
356
367
459
811
943
975
976
977
978
1013
%
m/z

Compound 2

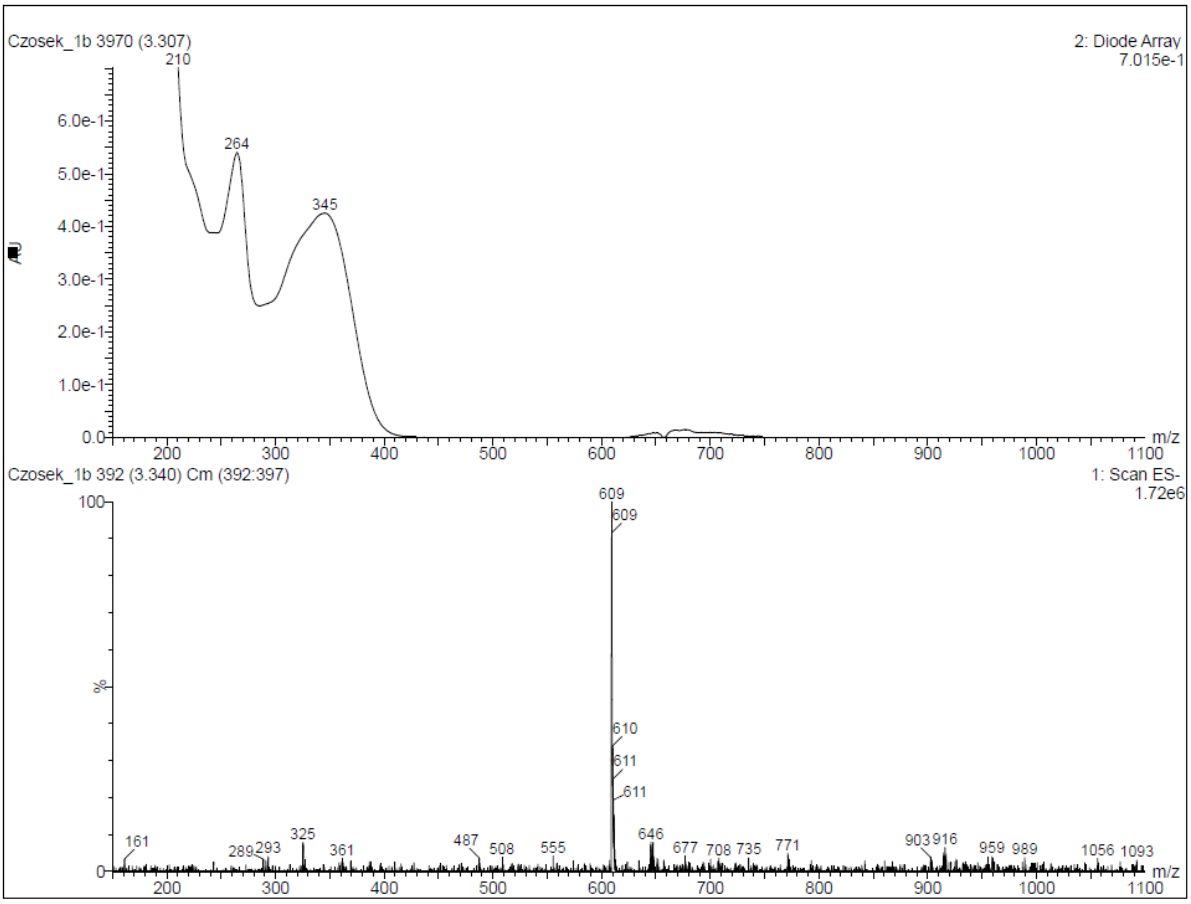

Czosek_1b 3970 (3.307)
2: Diode Array
7.015e-1
210
264
345
6.0e-1
5.0e-1
4.0e-1
3.0e-1
2.0e-1
1.0e-1
0.0
AU
200
300
400
500
600
700
800
900
1000
1100
m/z
Czosek_1b 392 (3.340) Cm (392:397)
1: Scan ES-
1.72e6
609
609
610
611
611
100
%
0
161
289
293
325
361
487
508
555
646
677
708
735
771
903
916
959
989
1056
1093

Compound 3

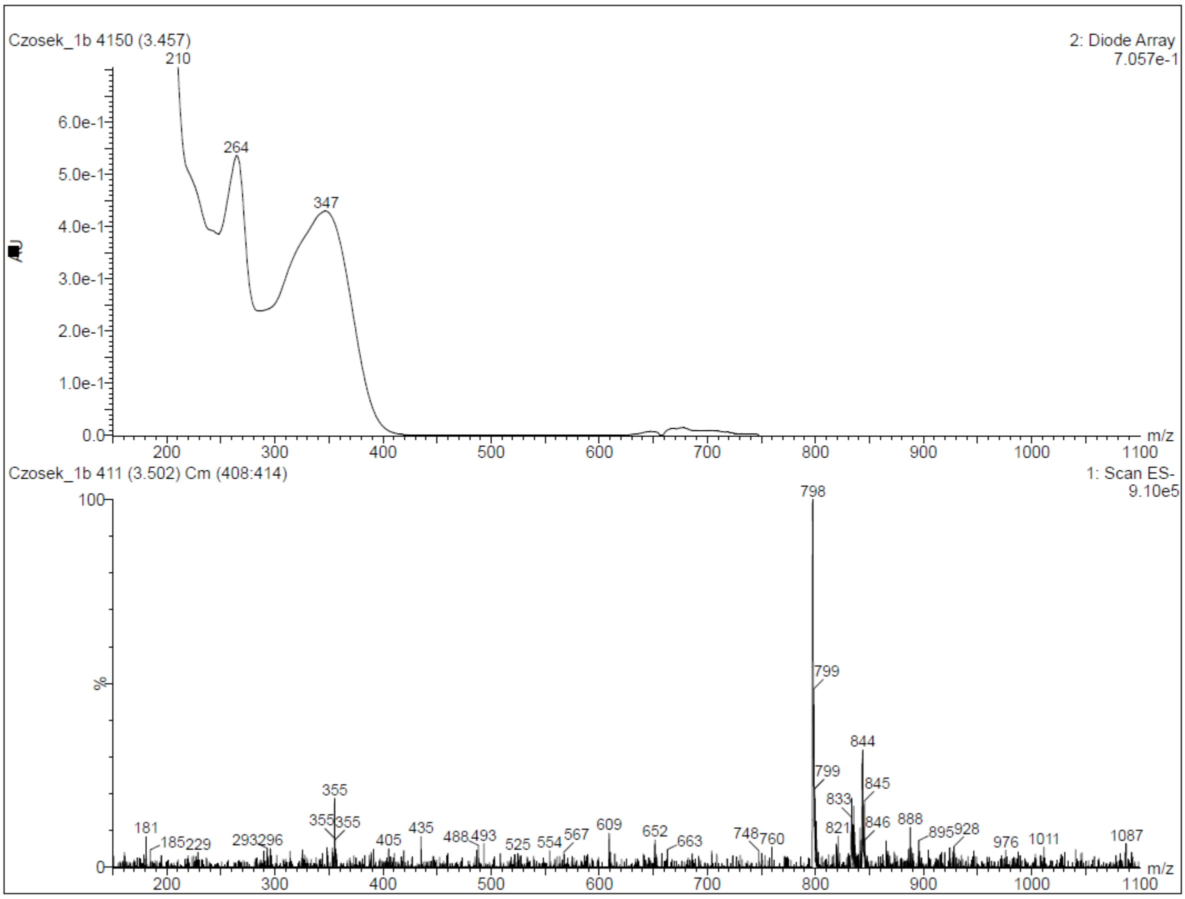

Czosek_1b 4150 (3.457)
2: Diode Array
7.057e-1
210
264
347
AU
Czosek_1b 411 (3.502) Cm (408:414)
1: Scan ES-
9.10e5
798
799
844
845
355
833
821
846
888
895
928
976
1011
1087
181
185
229
293
296
405
435
488
493
525
554
567
609
652
663
748
760
%
m/z

Compound 4

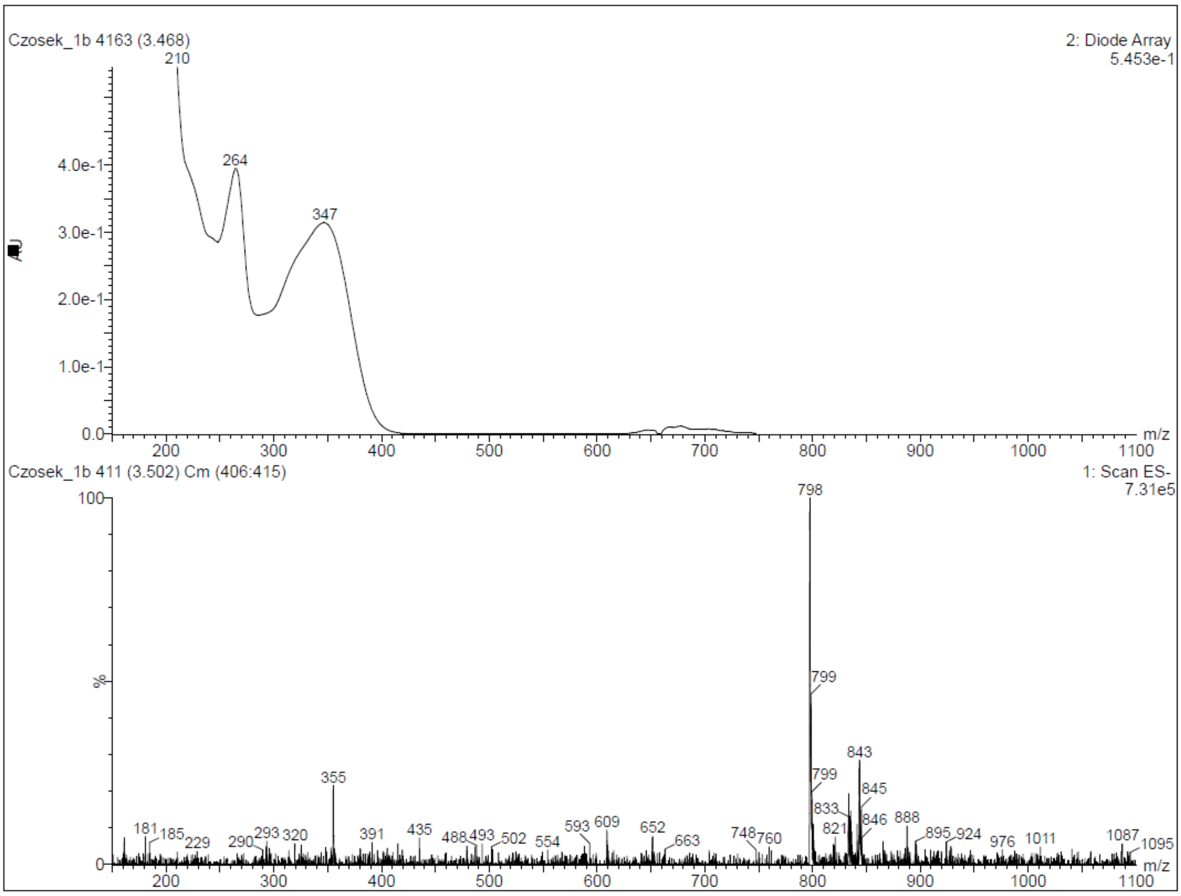
Czosek_1b 4163 (3.468)
2: Diode Array
5.453e-1
210
264
347
AU
4.0e-1
3.0e-1
2.0e-1
1.0e-1
0.0
200
300
400
500
600
700
800
900
1000
1100
m/z
Czosek_1b 411 (3.502) Cm (406:415)
1: Scan ES-
7.31e5
798
799
843
799
845
355
833
888
846
821
181
185
229
290
293
320
391
435
488
493
502
554
593
609
652
663
748
760
895
924
976
1011
1087
1095
100
%
0
m/z

Compound 5

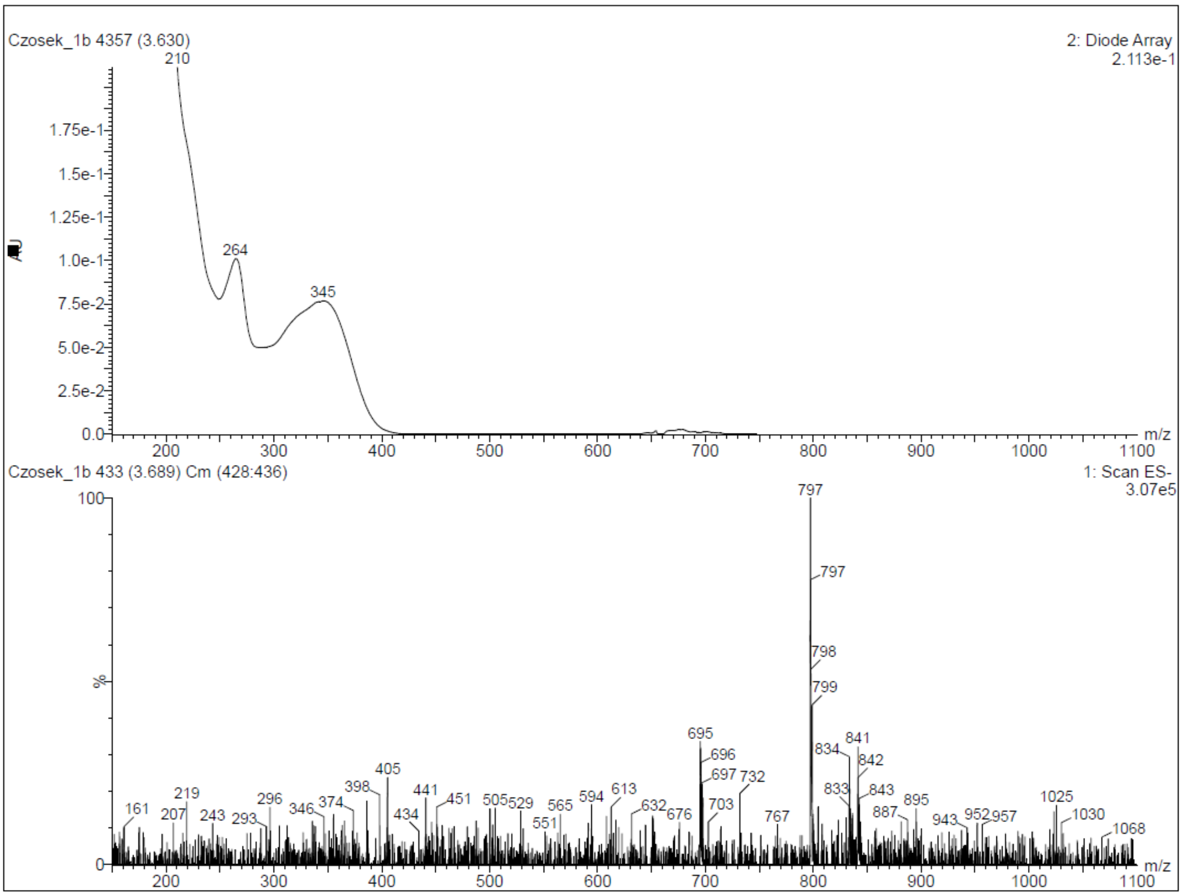

Czosek_1b 4357 (3.630)
2: Diode Array
2.113e-1
AU
210
264
345
1.75e-1
1.5e-1
1.25e-1
1.0e-1
7.5e-2
5.0e-2
2.5e-2
0.0
m/z
Czosek_1b 433 (3.689) Cm (428:436)
1: Scan ES-
3.07e5
797
798
799
695
696
697
732
834
841
842
833
843
%
100
0

Compound 6

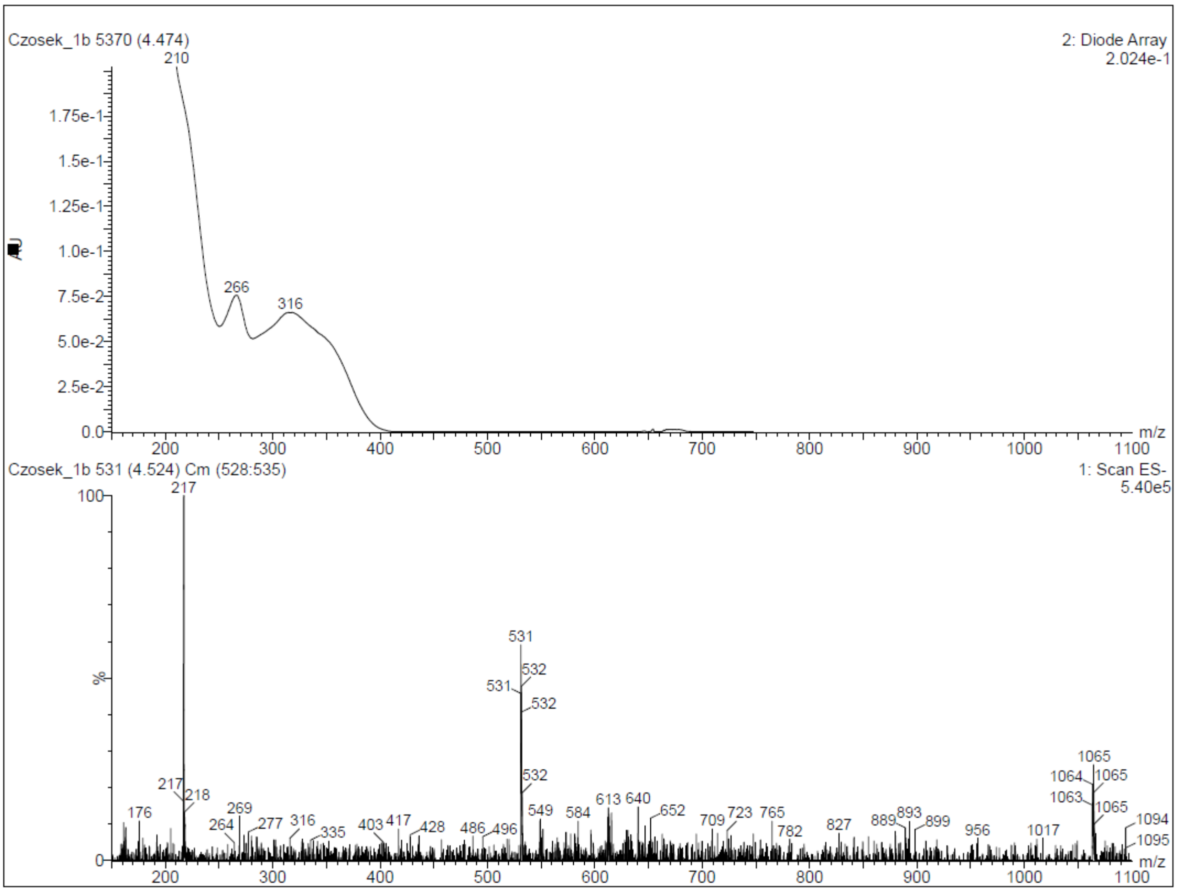

Czosek_1b 5370 (4.474)
2: Diode Array
2.024e-1
210
266
316
AU
1.75e-1
1.5e-1
1.25e-1
1.0e-1
7.5e-2
5.0e-2
2.5e-2
0.0
m/z
200
300
400
500
600
700
800
900
1000
1100
Czosek_1b 531 (4.524) Cm (528:535)
1: Scan ES-
5.40e5
100
217
531
532
531
532
532
%
217
218
176
269
264
277
316
335
403
417
428
486
496
549
584
613
640
652
709
723
765
782
827
889
893
899
956
1017
1065
1064
1065
1063
1065
1094
1095
0
m/z

Compound 7

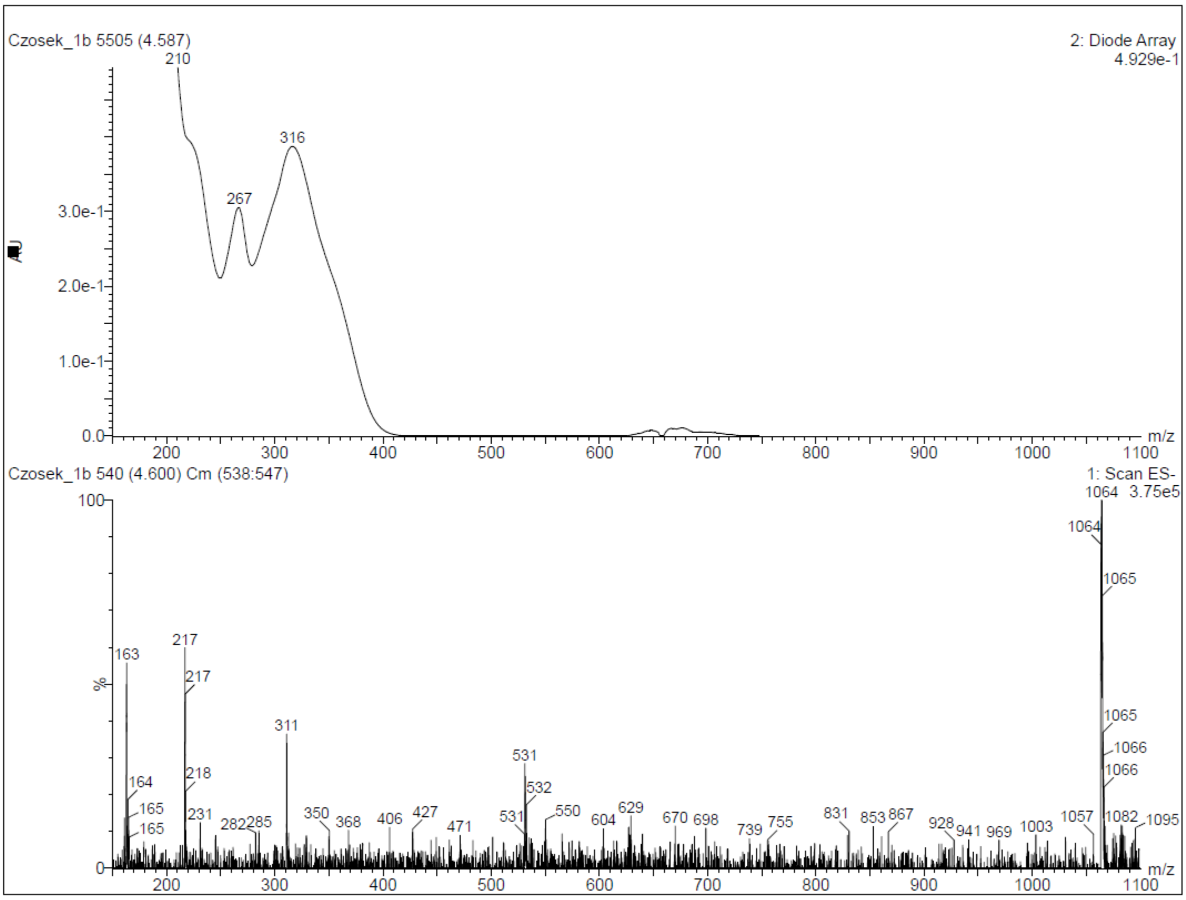
Czosek_1b 5505 (4.587)
2: Diode Array
4.929e-1
210
316
267
AU
m/z
Czosek_1b 540 (4.600) Cm (538:547)
1: Scan ES-
1064 3.75e5
%
m/z

Compound 8

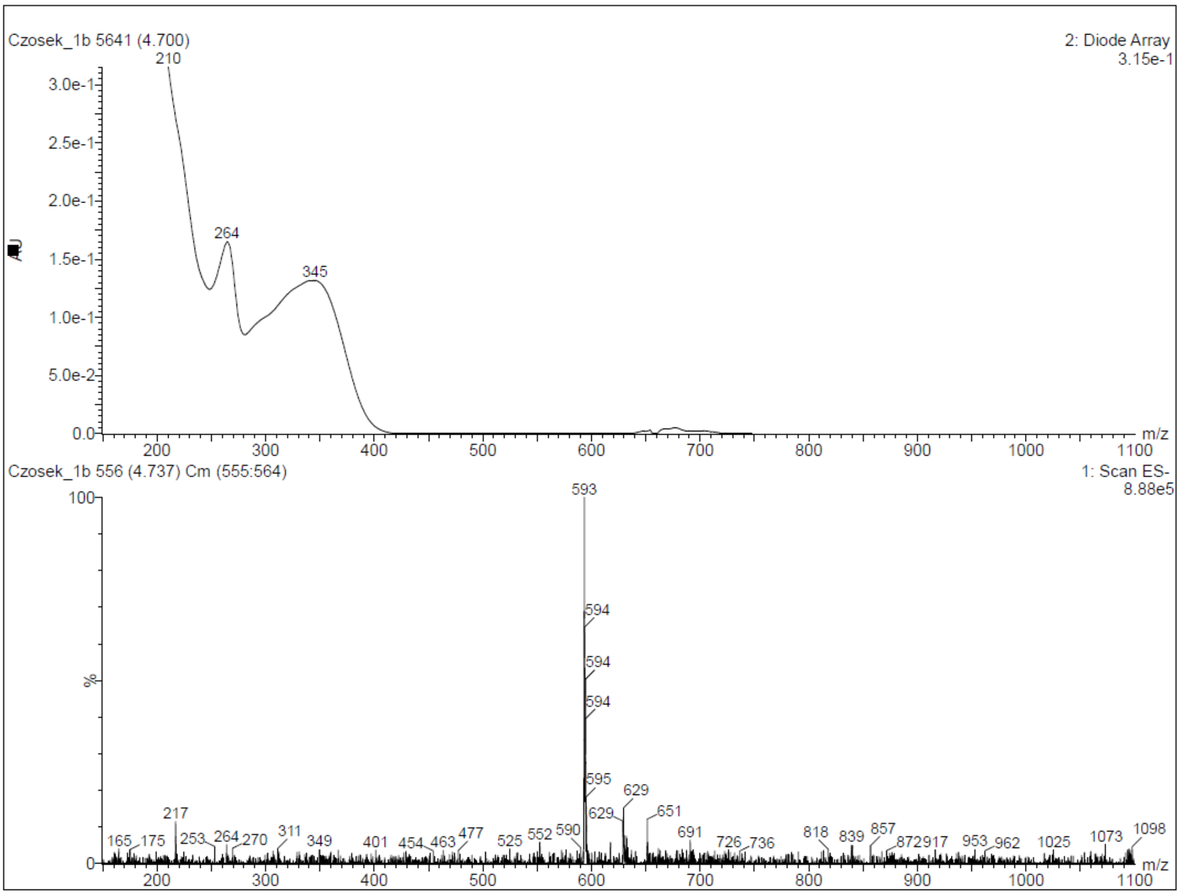

Czosek_1b 5641 (4.700)
2: Diode Array
3.15e-1
AU
m/z
Czosek_1b 556 (4.737) Cm (555:564)
1: Scan ES-
8.88e5
%
m/z

Compound 9

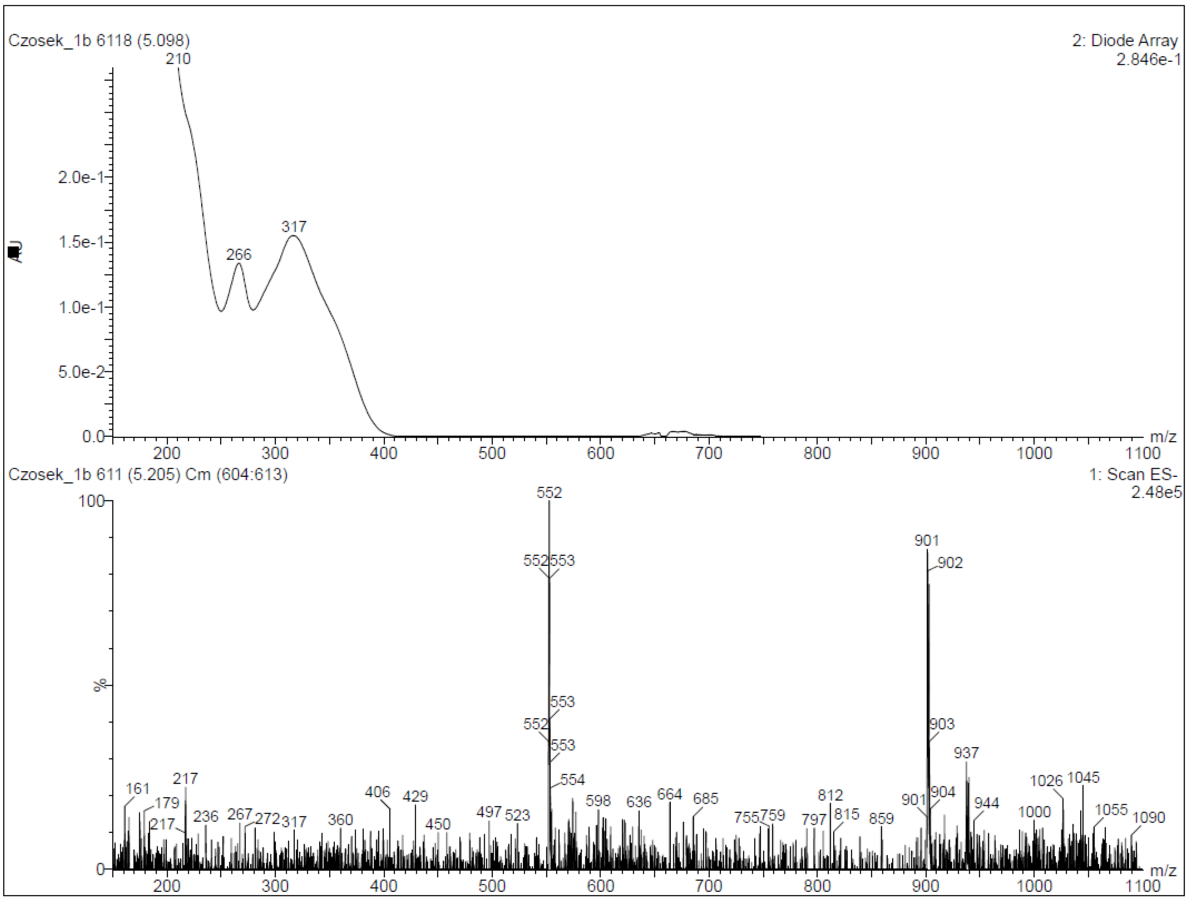
Czosek_1b 6118 (5.098)
2: Diode Array
2.846e-1
210
266
317
AU
m/z
Czosek_1b 611 (5.205) Cm (604:613)
1: Scan ES-
2.48e5
552
901
902
%
m/z

Compound 10

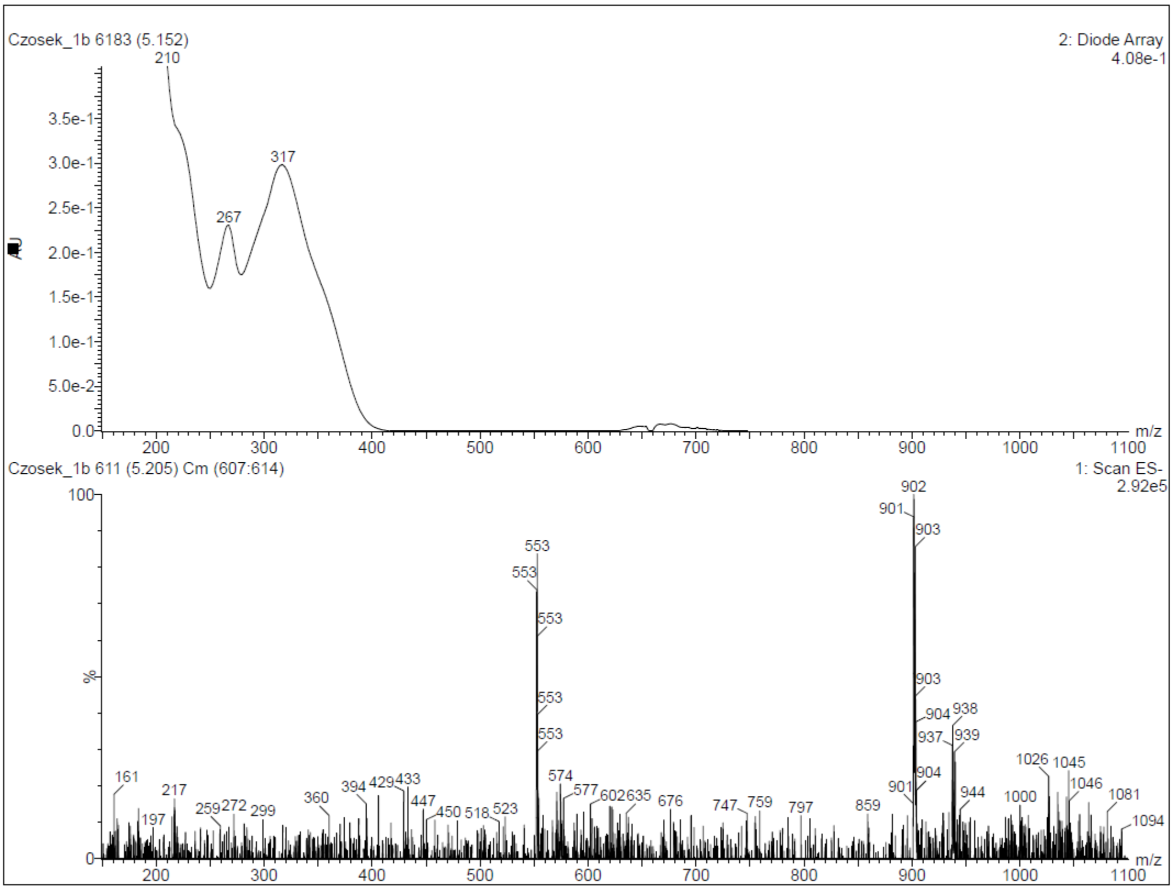

Czosek_1b 6183 (5.152)
2: Diode Array
4.08e-1
210
317
267
AU
3.5e-1
3.0e-1
2.5e-1
2.0e-1
1.5e-1
1.0e-1
5.0e-2
0.0
m/z
200
300
400
500
600
700
800
900
1000
1100
Czosek_1b 611 (5.205) Cm (607:614)
1: Scan ES-
2.92e5
100
%
0
902
901
903
553
553
553
553
553
903
904
938
937
939
904
901
944
161
217
197
259
272
299
360
394
429
433
447
450
518
523
574
577
602
635
676
747
759
797
859
1026
1045
1046
1000
1081
1094

Compound 11

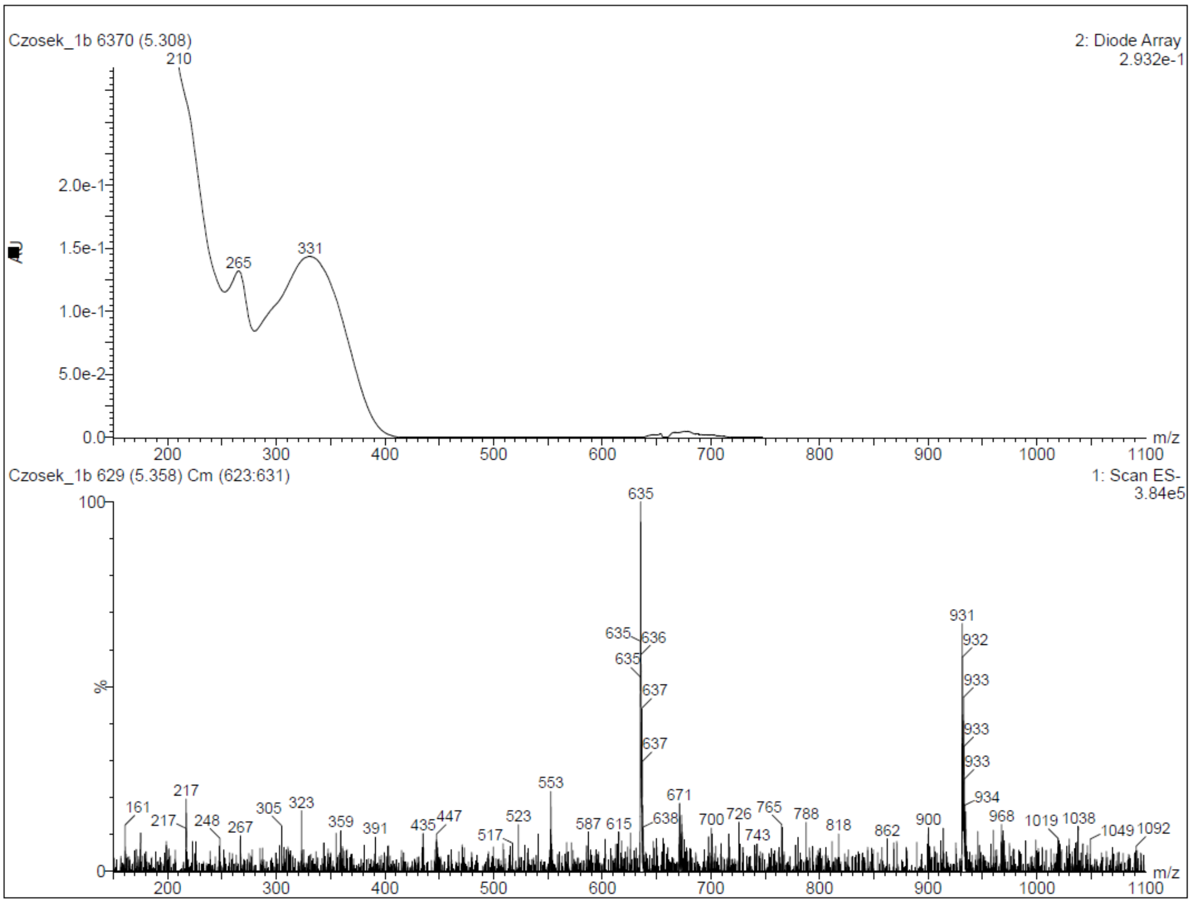
Czosek_1b 6370 (5.308)
2: Diode Array
2.932e-1
210
265
331
AU
m/z
Czosek_1b 629 (5.358) Cm (623:631)
1: Scan ES-
3.84e5
635
931
553
%
m/z

Compound 12

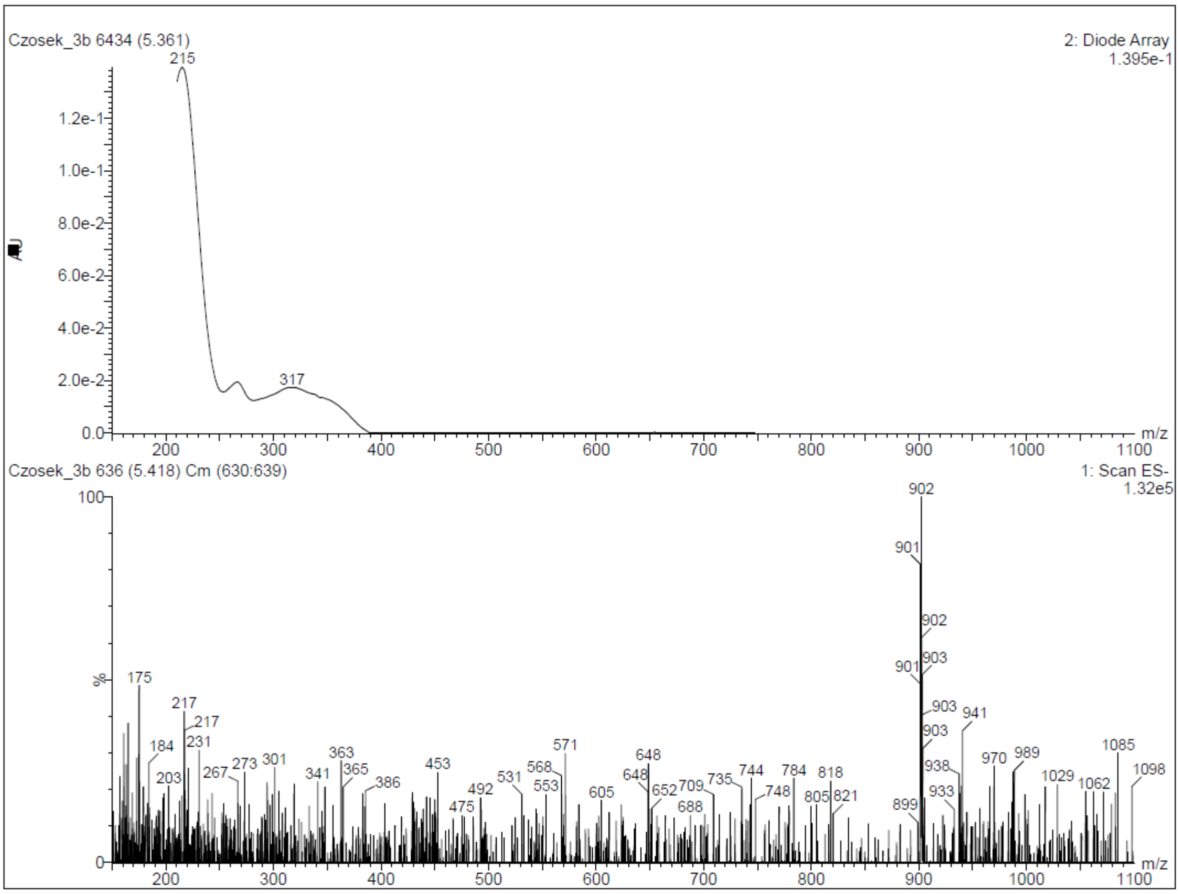
Czosek_3b 6434 (5.361)
2: Diode Array
1.395e-1
AU
215
317
m/z
Czosek_3b 636 (5.418) Cm (630:639)
1: Scan ES-
1.32e5
%
902
901
903
941
1085
1098
m/z

Compound 13

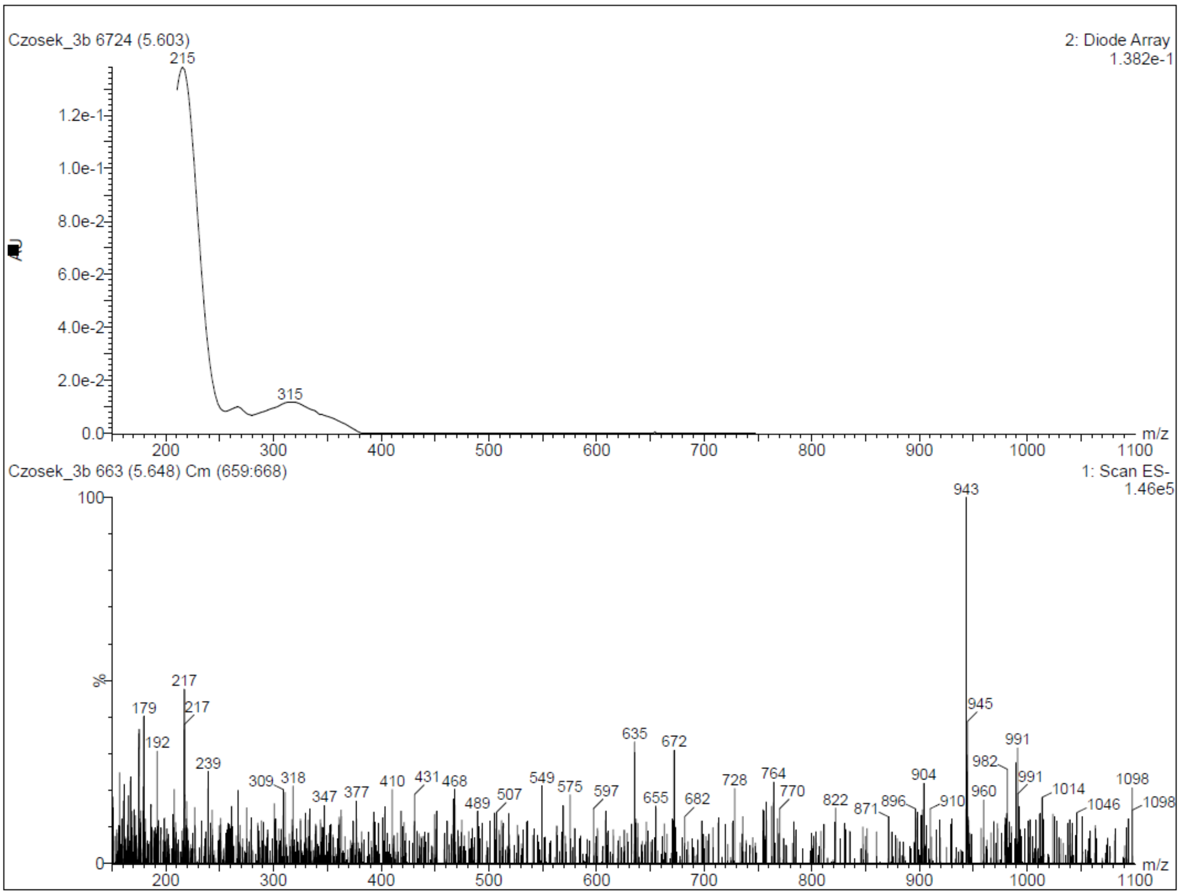

Czosek_3b 6724 (5.603)
2: Diode Array
1.382e-1
215
315
AU
m/z
Czosek_3b 663 (5.648) Cm (659:668)
1: Scan ES-
1.46e5
943
945
217
179
192
239
309
318
347
377
410
431
468
489
507
549
575
597
635
655
672
682
728
764
770
822
871
896
904
910
960
982
991
1014
1046
1098
%
m/z

Compound 14

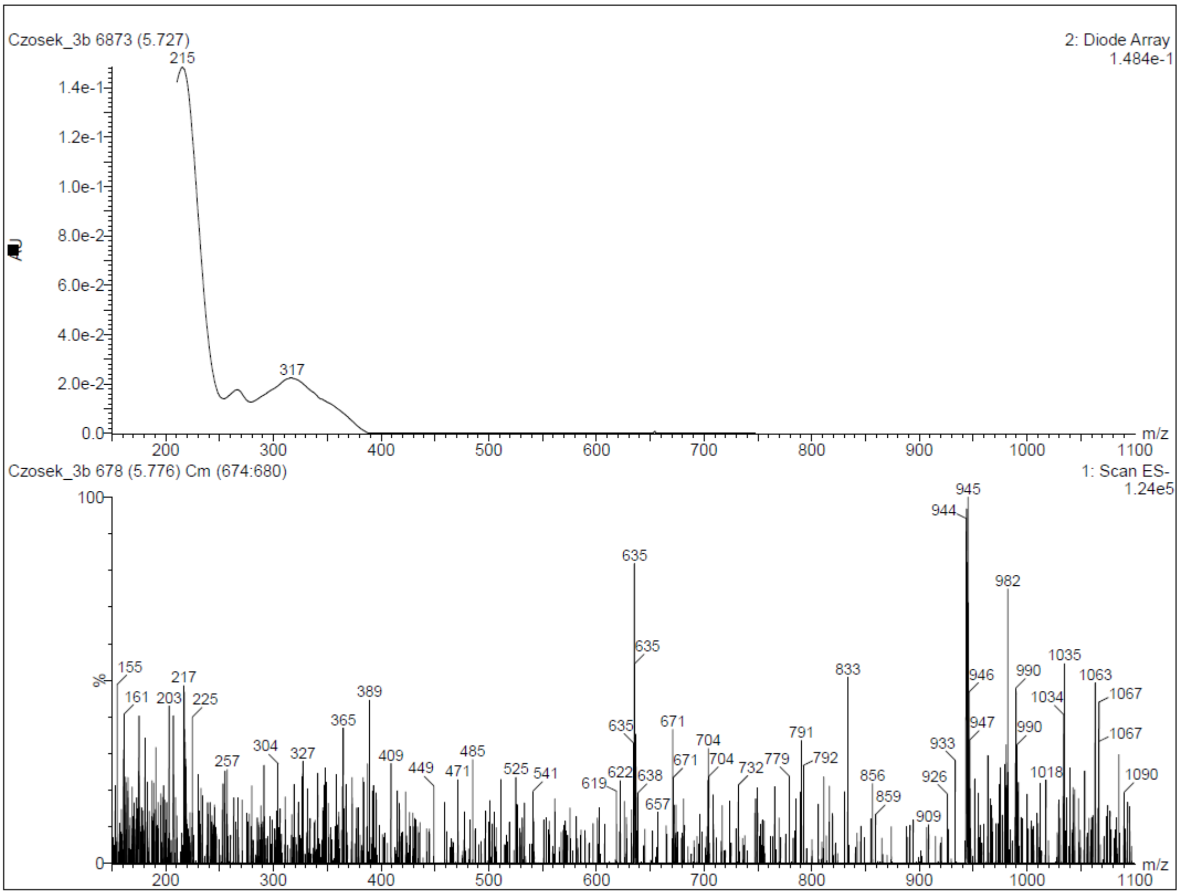
Czosek_3b 6873 (5.727)
2: Diode Array
1.484e-1
AU
m/z
Czosek_3b 678 (5.776) Cm (674:680)
1: Scan ES-
1.24e5
%
m/z

Compound 15

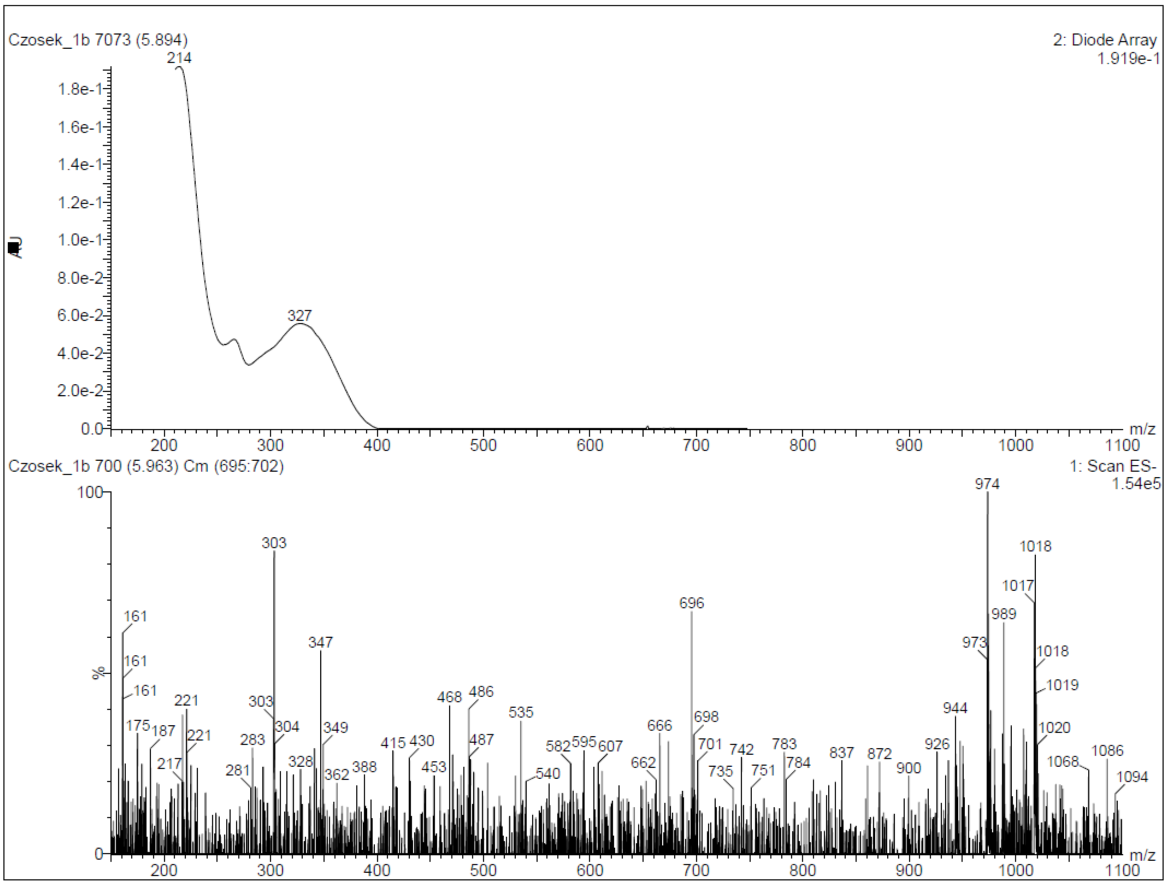

Czosek_1b 7073 (5.894)
2: Diode Array
1.919e-1
214
327
AU
m/z
Czosek_1b 700 (5.963) Cm (695:702)
1: Scan ES-
1.54e5
974
303
1018
1017
696
161
989
347
973
1018
1019
%
m/z

Compound 16
